# Unravelling population structure heterogeneity within the genome of the malaria vector *Anopheles gambiae*

**DOI:** 10.1186/s12864-021-07722-y

**Published:** 2021-06-08

**Authors:** Melina Campos, Luisa D. P. Rona, Katie Willis, George K. Christophides, Robert M. MacCallum

**Affiliations:** 1 Department of Life Sciences, Imperial College London, London, UK; 2 Department of Cell Biology, Embryology and Genetics, Federal University of Santa Catarina (UFSC), Florianópolis, Brazil; 3 National Institute of Science and Technology in Molecular Entomology, National Council for Scientific and Technological Development (INCT-EM, CNPq), Rio de Janeiro, Brazil

**Keywords:** T-SNE, Population genetics, Chromosomal inversions, Whole-genome analysis, Malaria, Visualization method

## Abstract

**Background:**

Whole genome re-sequencing provides powerful data for population genomic studies, allowing robust inferences of population structure, gene flow and evolutionary history. For the major malaria vector in Africa, *Anopheles gambiae*, other genetic aspects such as selection and adaptation are also important. In the present study, we explore population genetic variation from genome-wide sequencing of 765 *An. gambiae* and *An. coluzzii* specimens collected from across Africa. We used t-SNE, a recently popularized dimensionality reduction method, to create a 2D-map of *An. gambiae* and *An. coluzzii* genes that reflect their population structure similarities.

**Results:**

The map allows intuitive navigation among genes distributed throughout the so-called “mainland” and numerous surrounding “island-like” gene clusters. These gene clusters of various sizes correspond predominantly to low recombination genomic regions such as inversions and centromeres, and also to recent selective sweeps. Because this mosquito species complex has been studied extensively, we were able to support our interpretations with previously published findings. Several novel observations and hypotheses are also made, including selective sweeps and a multi-locus selection event in Guinea-Bissau, a known intense hybridization zone between *An. gambiae* and *An. coluzzii*.

**Conclusions:**

Our results present a rich dataset that could be utilized in functional investigations aiming to shed light onto *An. gambiae s.l* genome evolution and eventual speciation. In addition, the methodology presented here can be used to further characterize other species not so well studied as *An. gambiae*, shortening the time required to progress from field sampling to the identification of genes and genomic regions under unique evolutionary processes.

**Supplementary Information:**

The online version contains supplementary material available at 10.1186/s12864-021-07722-y.

## Background

*An. gambiae* was originally described as a single mosquito taxon in 1902 by Giles, but was later identified as a complex composed of at least eight morphologically indistinguishable yet molecularly divergent sibling species, collectively referred to as *An. gambiae s.l.* [1–4]. Polytene chromosomes studies revealed an abundance of paracentric inversion polymorphisms, of which a few fixed inversions distinguish six of these species [5, 6]. A recently separated species of the complex, *An. gambiae s.s* and *An. coluzzii* (formerly S and M molecular forms, respectively), share inversion karyotypes and were originally distinguished by species-specific Single Nucleotide Polymorphisms (SNPs) in a ribosomal locus [7].

Several studies have focused on understanding the origin and evolution of inversions as well as their association with speciation and local adaptation [8–10]. Most notably, the frequencies of the largest and most geographically distributed inversions in the second chromosome of *An. gambiae* (2L*a* and 2R*b*) have been shown to correlate with an African aridity cline [5, 11, 12]. Causal validation of this correlation was performed by phenotypic experiments with characterized laboratory colonies [13, 14] and controlled karyotype crosses [8].

Population genomic studies were accelerated after the publication of the first complete genome sequence of an *An. gambiae* colony containing both the M and S molecular forms [15]. Using genome re-sequencing or SNP microarrays, genomic regions and loci were identified that have diverged between *An. gambiae s.s.* and *An. coluzzii* [16–19], or between populations with differing insecticide resistance phenotypes [20, 21]. The latest most ambitious population study was the re-sequencing of nearly 1000 genomes of *An. gambiae* s.s. and *An. coluzzii*, which revealed high levels of genetic diversity in natural populations and provided a platform to explore further genetic factors in this important malaria vector (the *An. gambiae* 1000 Genomes Consortium, Ag1000G henceforth). The study surveyed genomic population structure heterogeneity by analysing 100 kbp windows and identified four types of evolutionary dynamics, whereby population structure is governed by species, geography, 2L*a* inversion genotype or 2R*b* inversion genotype [22].

Here, we perform an analysis of local population structure at a finer-grained genomic resolution. We describe a new method to visualize and expedite the analysis of data from genome re-sequencing projects that sample hundreds to thousands of individuals from diverse collection sites. This method uses the t-Distributed Stochastic Neighbor Embedding (t-SNE [23]) visualization algorithm to reduce the high-dimensional inter-individual genetic distance information extracted from the Ag1000G project into a 2D map-like representation, such that one can visualize clusters of genes that may have been subject to the same or similar evolutionary forces. We show that many regions of the gene map are strongly linked to genomic location, particularly influenced by speciation islands [17], chromosomal inversions and biological function. Genes within recent selective sweeps are clearly demarcated on the gene map. Unlike existing methods for quantifying selection, this approach does not require a fully assembled reference genome. Our study complements previous approaches for genome-wide visualization of expression data [24] and provides a new way to explore population genomics data, which can be easily applicable to any organism and accelerate the discovery of novel genomic features shaping the species.

## Results & discussion

### Dataset

Using data from the Ag1000G Project (phase 1 AR3 data release; https://www.malariagen.net/projects/ag1000) carried out by the Malaria Genomic Epidemiology Network (MalariaGEN) [22], SNPs were obtained for each gene. The dataset contains 765 samples (619 *An. gambiae s.s.*, 132 *An. coluzzii* and 14 hybrids) from 8 African countries comprising Angola, Burkina Faso, Cameroon, Gabon, Guinea, Guinea-Bissau, Kenya and Uganda. Mosquitoes were collected between 2009 and 2012, except the Gabon samples which were collected in 2000. Samples from Burkina Faso were separated between species, *An. gambiae s.s* and *An. coluzzii*. Due to high frequency of hybrids [25, 26], Guinea-Bissau was considered as a single mixed population of the two sibling species. SNPs in non-coding regions were removed and SNP numbers were counted after filtering (S2 Table). Only SNPs within exons were used in this study to minimize the confounding effects of nested genes. In total 11,318 genes were included for downstream analysis corresponding to 90.14% of *An. gambiae* genes in VectorBase [27] gene set Agam4.2 (S2 Table). These genes are distributed throughout all 4 chromosomal arms, i.e. 2R, 2L, 3R, 3L and X chromosome (S2 Table). No single mosquito or gene had a substantial (> 4%) amount of missing data (S3 Table).

### A gene-resolution map of *An. gambiae s.s.* and *An. coluzzii* populations

**Fig. 1:**
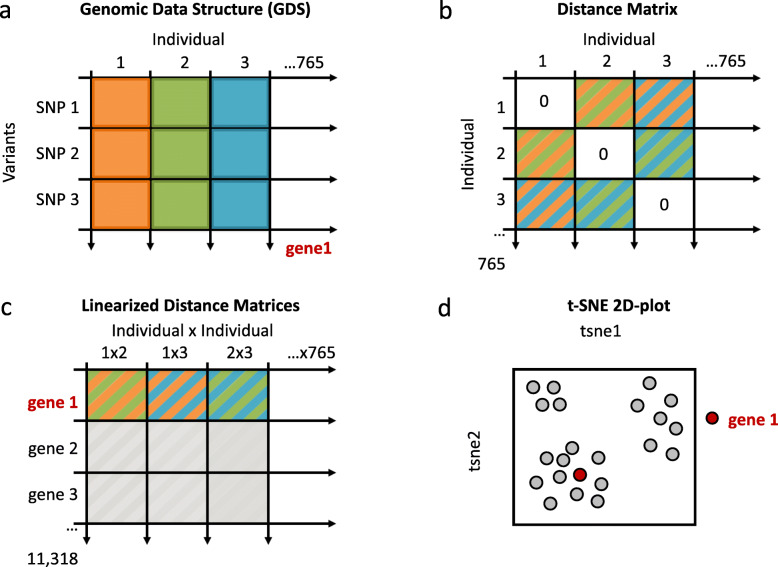
Methodology outline. Schematic figure of the methodology used, from variant call format (VCF) files to 2D-plot of t-SNE. **a** Genomic Data Structure (GDS), converted file from VCF, for each gene. Number of variants may vary for a constant number of individuals (*N* = 765). **b** Individual by individual distance matrix calculated for each gene (765 × 765; 292,230 unique distances). **c** Table containing linearized distance matrix values for 11,318 genes (11,318 × 292,230 values). **d** t-SNE 2D-plot using the table with linearized distances for all genes

Inter-individual genetic distance matrices (765 × 764 / 2 = 292,230 individual pairs) were calculated for each of the 11,318 genes (methods summarized in Fig. 1). The distance matrices were linearized and combined for all genes into one large matrix (11,318 genes × 292,230 individual pairs). The t-SNE algorithm was used to flatten this high-dimensional data into a representation depicting genes in two dimensions. The goal of this dimensionality reduction is to preserve as much of the high dimensional population structure information in the low dimensional representation as possible. The graph-based t-SNE algorithm differs from matrix factorization methods such as principal components analysis (PCA) and multidimensional scaling (MDS) in that it is concerned primarily with local relationships between genes (genes with highly similar population structures) and is able to flatten complex manifolds to some extent, although global relationships are not usually reliably represented. Our approach has similarities to the TREESPACE algorithm for tree clustering [28]. However, the dimension-reducing method used by TREESPACE, MDS, does not scale well to large datasets [29] and over-emphasises long-range distances as discussed above.

**Fig. 2:**
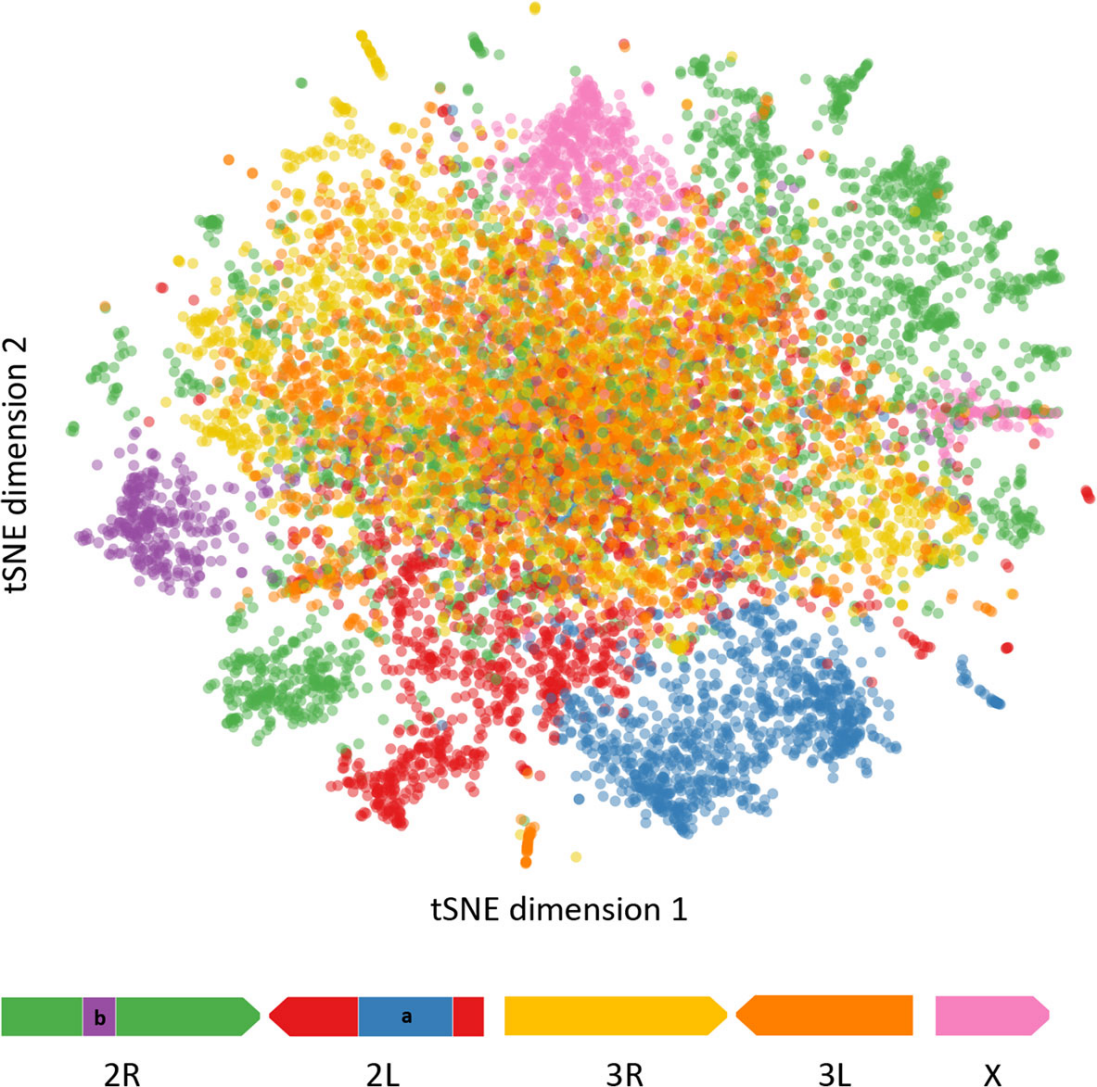
Scatter plot of 11,318 genes of *Anopheles gambiae s.l.* Dimensional reduction by t-SNE using SNP data for 11,318 genes of *A. gambiae s.s.*and *A. coluzzii*. Each dot is a gene, which was colored according to chromosome arm location and/or main known inversion in the *A. gambiae* genome. Below is a schematic representation of the *A. gambiae* genome

The resulting all-gene t-SNE is shown in Fig. 2, where genes are colored by chromosome arm and two previously described chromosomal inversions: 2L*a* and 2R*b* [5, 30]. Approximately 6000 genes from a mix of chromosomes form a large homogenous central region, while surrounding clusters of various sizes appear to be defined by chromosomal arm and inversions, i.e. genes on the same chromosomal arm or within the same inversion tend to be co-located on the t-SNE.

Because t-SNE has a random initialization step, each invocation of the algorithm could produce a different result. The results of 30 independent replicate t-SNE mappings (S1, S2 and S3 Figs; S4 Table; and also interactively via the web interface at https://vigilab.shinyapps.io/anopheles/) show that, overall, the dense central region and the large and smaller peripheral clusters are consistent in terms of gene content, though their relative positions are more variable. Henceforth in this article, “consistent clustering” or “consistently clustered” refers to highly reproducible cluster membership across the 30 plots. The variability of global cluster arrangement between the repeated t-SNE plots clearly illustrates the limitations of the method with respect to the accurate reproduction of long-range relationships. Thus, we warn against trying to interpret the high-level structure of the plots.

**Fig. 3:**
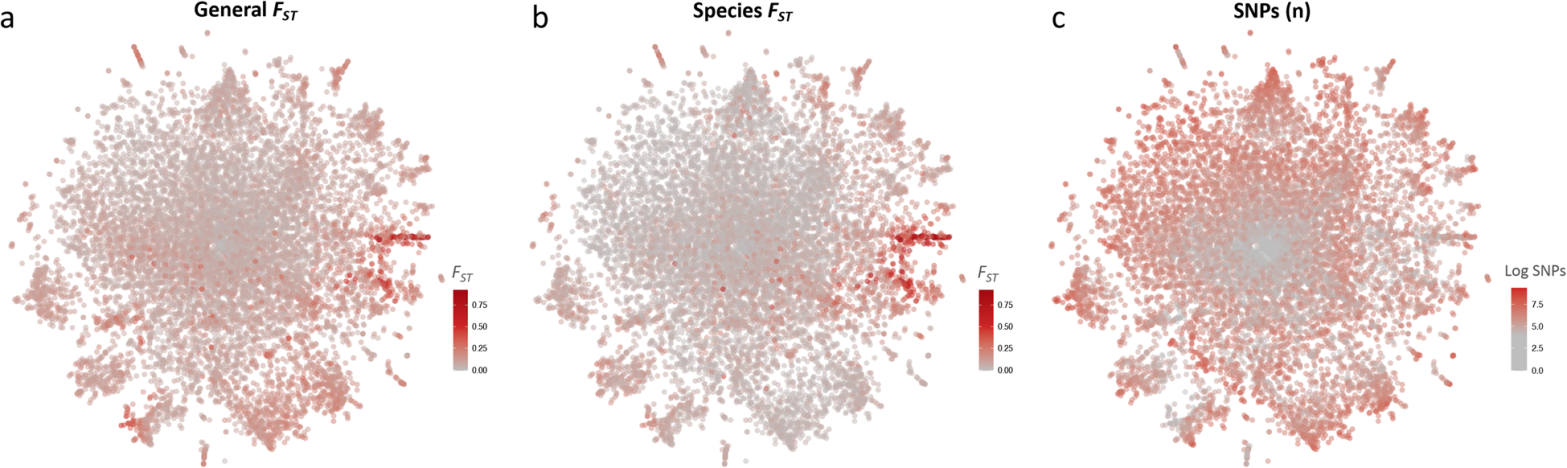
Ranked distribution of *F*_*ST*_ values and number of SNPs for 11,318 genes of *Anopheles gambiae s.l.* **a** General *F*_*ST*_ values based on mean overall *F*_*ST*_ values between all 9 populations; **b** Species *F*_*ST*_ values calculated using *A. gambiae s.s* vs *A. coluzzii* subsets. **c** Number of SNPs for each gene

Although the layout of genes on t-SNE is driven by inter-*individual* genetic distances, it is informative to use *population*-based genetic measures such as the fixation index (*F*_*ST*_) as an aid to interpret the map. *General F*_*ST*_, which quantifies the average divergence between all pairs of populations defined by country and sibling species, is high (*F*_*ST*_ > 0.2) in most of the outer clusters (Fig. 3a; S5 Table).

There is also a clear global trend for an increased number of SNPs per gene from the center to the periphery of the map (Fig. 3c). This trend is also seen at a smaller scale within major peripheral clusters. Thus, a broad interpretation of the map is that genes with undifferentiated SNPs tend to be located in the center, while more differentiated genes are found in several distinct, peripheral clusters (Fig. 3a, b).

### X chromosome and speciation islands

In the *An. gambiae s.l. complex*, a “speciation continuum” is observed, i.e. species undergo heterogenous gene flow [31, 32], genomic introgression [25, 33, 34], and uncertain boundaries [35, 36]. Most of the species within this complex were first distinguished by inter-species hybridization resulting in sterile male progeny or by the presence of fixed chromosomal inversions [2, 4]. Centromeric regions of chromosomes have been demonstrated to contain high levels of differentiation and often described as “islands of speciation” [16, 17]. One of these, a region on the X chromosome, has been especially associated with the speciation process [36, 37]. According to Fontaine et al. [36], a 15 Mb region of the chromosome X reveals the ‘true’ species tree between *An. gambiae*, *An. arabiensis* and *An. melas*, while autosomes are misleading due to extensive historical introgression between species. Similarly, Lee et al. [38] showed that markers on the X chromosome have greater diagnostic power than those on autosomes for divergence between *An. gambiae* and *An. coluzzii*. Later, Aboagye-Antwi et al. [37] demonstrated that the X chromosome island only plays a key role in assortative mating between *An. gambiae* and *An. coluzzii*.

**Fig. 4:**
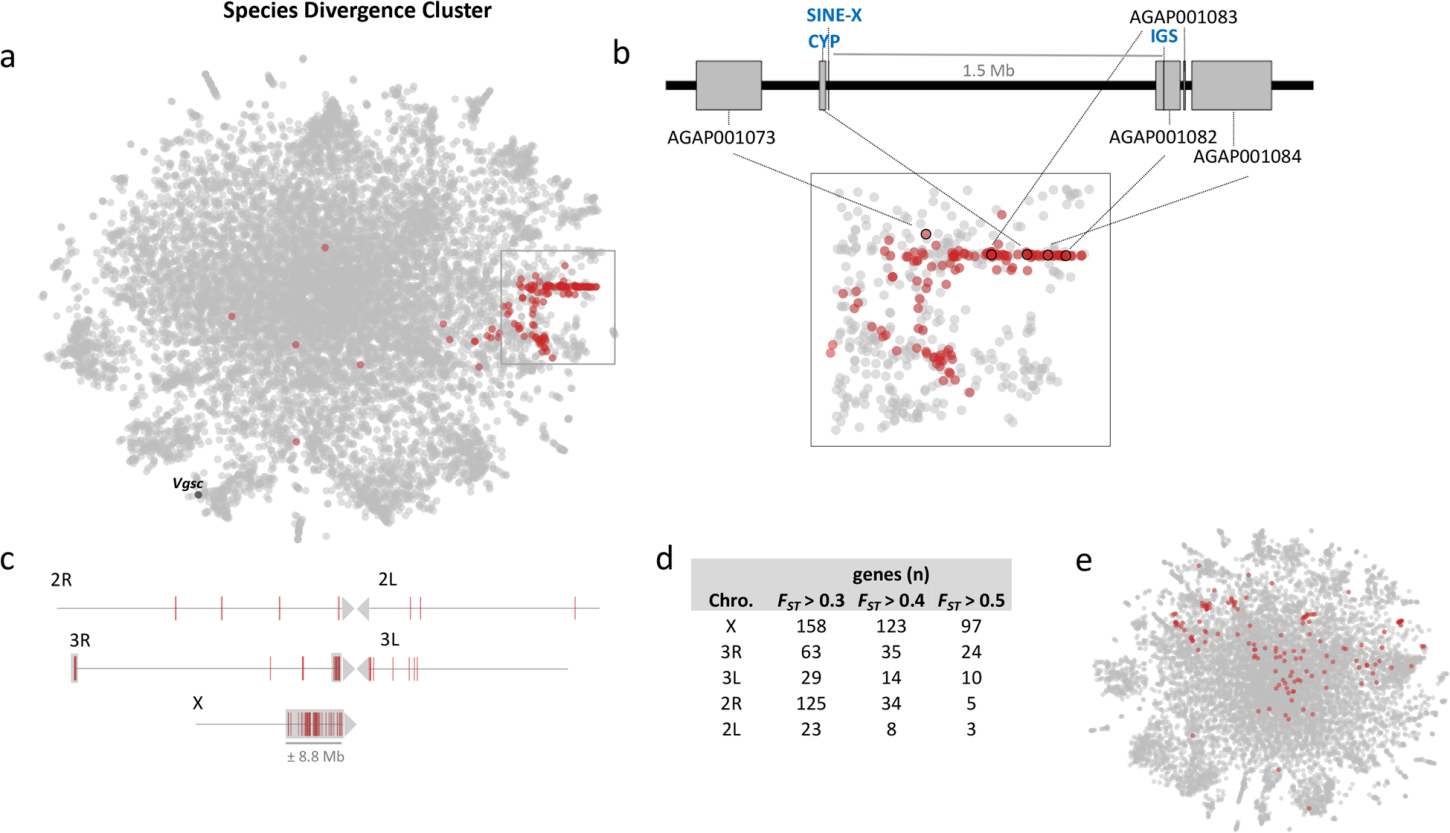
Sibling species differentiation. **a** t-SNE plot highlighting genes that show two criteria: Species *F*_*ST*_ higher than General *F*_*ST*_ and Species *F*_*ST*_ values higher than 0.5 (red). These genes are largely located either on the centromeric half of the X chromosome or close to the 3R centromere. In black, gene *Vgsc* (AGAP004707)*.* **b** Enlargement of the ‘speciation cluster’ showing neighboring genes (AGAP001073, AGAP001082, AGAP001083, AGAP001084) of the main diagnostic markers of *A. gambiae* and *A. coluzzii*: IGS, SINE200 and *CYP* (in blue). **c** Schematic representation of gene starting point for every gene selected in **a**; shaded area corresponds to previously described islands of speciation. **d** Table containing the distribution of genes through chromosome arms in the ‘speciation cluster’. **e** Scatter plot of the same 11,318 genes of *A. gambiae* genome using only *An. gambiae s.s.* samples, showing loss of a defined ‘speciation cluster’

Genetic differences between the two sibling species included in the dataset analysed here, namely *An. gambiae s.s.* and *An. coluzzii*, contribute to the clustering in the t-SNE of genes that may be relevant to speciation (“species divergence cluster”, Fig. 4). Genes with high *Species F*_*ST*_ are located on the right-hand side of the plot (*F*_*ST*_ > 0.5) and are predominantly located either on the centromeric half of the X chromosome or close to the 3R centromere (Fig. 4a, b), which is consistent with previous studies that have detected high level of divergence between *An. gambiae s.s.* and *An. coluzzii* on speciation islands [16, 17, 39, 40]. Additionally, several high *Species F*_*ST*_ genes from other autosomal locations are consistently co-clustered with these X and 3R centromeric genes. If a t-SNE is made with only samples from one species (*An. gambiae s.s.*) the co-clustering of these genes is lost (Fig. 4e).

Thus, the t-SNE provides a visual indication of the genomic extent of “islands of speciation”. Starting with genes close to known X-linked species-diagnostic markers (e.g. the intergenic spacer (IGS) of the multicopy ribosomal DNA [41, 42] and the *An. coluzzii*-specific SINE (short interspersed element) insertion [43], one can identify autosomal genes that are consistently co-clustered with them (Fig. 4; S6 Table). One such gene, OBP41 (odorant binding protein 41; AGAP005182; non-centromeric 2L; *Species F*_*ST*_ 0.53) is interesting because it is highly expressed in ovaries 48 h after a blood meal [44, 45]. The protein product of this gene may, like other atypical-type ovary-expressed OBPs, be present in the eggshell and have a role in sperm chemotaxis [46]. Also, co-clustered with OBP41 and genes of X-linked speciation is AGAP001820 (genomic location: 2R*j* inversion; *Species F*_*ST*_ 0.18). This gene is a one-to-one ortholog of *Drosophila melanogaster* Helicase 89B (Hel89B) which encodes a DNA-binding protein that acts as a chromatin regulator. The high level of expression of AGAP001820 in the testis mirrors the ovarian expression of OBP41 and is likewise suggestive of a role of this gene in speciation [44]. Furthermore, two odorant receptor genes Or37 (AGAP002126; chromosome 2R; *Species F*_*ST*_ 0.26) and Or60 (AGAP011979; 3L; *Species F*_*ST*_ 0.53) and TEP3 (thioester-containing protein 3, AGAP010816; 3L; *Species F*_*ST*_ 0.28) are consistently co-clustered. The odorant receptor genes have shown sex-biased expression in *An. gambiae*, where Or37 was differentially expressed in male reproductive tissues [47] and Or60 in females after a blood meal [45]. The immunity gene, TEP3, has been previously identified as having long-range LD with speciation island regions and highlighted as differentiated between *A. gambiae* and *A*. *coluzzii* [48, 49]. Thus, several genes that may have either driven the speciation process, or be directly downstream of it, have been identified easily using this visual tool.

Recent adaptive introgression on the left arm of chromosome 2 (2L) was repeatedly detected in natural populations conferring homogenization of autosomal genomic islands [31, 33, 50]. For example, under strong selective pressure by insecticides, *An. coluzzii* inherited the entire *An. gambiae*-associated large centromeric region of chromosome 2L 2L, where the voltage-gated sodium channel (*Vgsc,* AgamP4 gene ID = AGAP004707) gene that confers insecticide resistance is found [33, 34]. Homogenization of this genomic region via introgression therefore explains why centromeric genes from 2L are not highlighted in Fig. 4a.

### Chromosomal inversions

As seen in Fig. 2, genes located within the 2L*a* and 2R*b* inversions form two well delineated clusters (blue and purple clusters in Fig. 2, respectively). The distinct population structures for the genes in these clusters is expected due to the much-reduced recombination rate within inversions in heterokaryotypes that reduces gene flow between homokaryotypes [51]. Chromosomal inversions are the result of reversed reinsertion of two break points, and like any other type of mutation, evolve under selection and random drift [51]. These two inversions in the second chromosome of *An. gambiae* have a broad geographic distribution and their frequency as the degree of aridity increases [5, 11]. Genomic resequencing of *An. gambiae* collected along the cline, has shown evidence of local adaptation i.e. environmental/ecological conditions maintain the cline inversion distribution [12, 52].

**Fig. 5:**
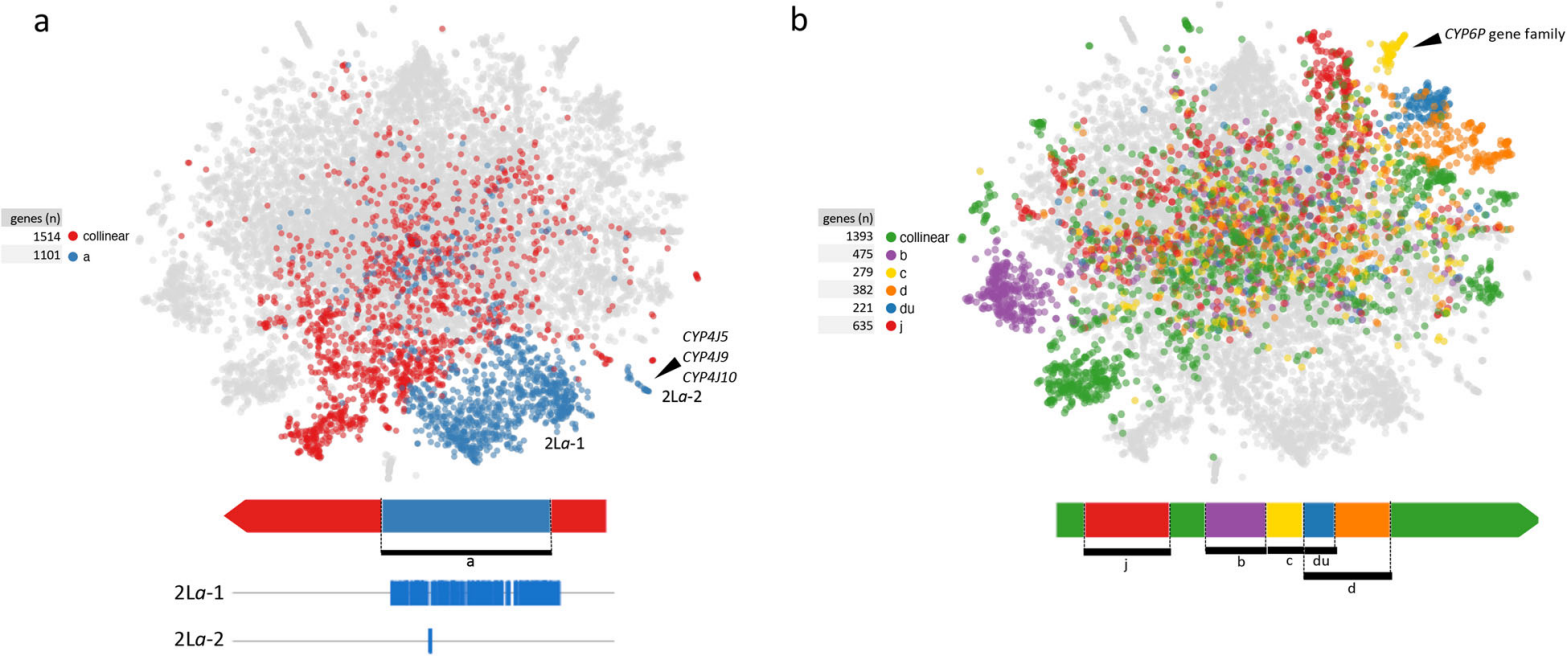
Chromosome 2 and its inversions. **a** t-SNE plot highlighting genes on the 2L chromosome arm, colors correspond to the genomic regions: outside inversion i.e. collinear (red), within inversion 2L*a* (blue). Within 2L*a* inversion: 2L*a*-1 showed as a large cluster and 2L*a*-2 as an small isolated cluster. **b** t-SNE plot highlighting genes on the 2R chromosome arm-colors correspond to the genomic region: outside inversions i.e. collinear (green), inversions and inversions overlap (*b*, *c*, *d*, *du*, *j*). Black arrows indicate key genes referenced in the text

In the present study, a total of 2615 genes located in the 2L chromosome arm were included, of which 1087 (41.5%) are within the 22 Mb 2L*a* inversion. In the dataset studied here, 75% of the individuals are homokaryotypes, including 43% 2L*a*-standard (+_a_/+_a_) and 32% 2L*a*-inverted (a/a). Genes mapped within the 2L*a* inversion formed one large cluster (2L*a*-1) and a small cluster (2L*a*-2) in the plot (Fig. 5a). The 2L*a*-2 cluster (or in some cases, just the subset of its 15 most distal genes) is present in all 30 replicate plots (S1 Fig) and is discussed in the selective sweeps section.

The other inversion, 2R*b*, is approximately 7.7 Mb long and consequently comprises a smaller number of genes (475 genes), although the gene density here is higher (2L*a*- 50 genes/Mb; 2R*b*- 61 genes/Mb) (Fig. 5b). In the present study, 62% of the individuals are homokaryotypes for 2R*b*-standard (+_b_/+_b_) and 17% homokaryotypes for 2R*b*-inverted (b/b). Together with 2R*b*, the other four common polymorphic inversions on this chromosome arm (*c*, *d*, *j*, and *u,* and overlapped *du*) are highlighted in Fig. 5b. The clustering of genes in the t-SNE largely follows the pattern of inversions and their overlaps. For example, where the 2R*u* inversion overlaps with 2R*d*, genes in this region (in blue in Fig. 5b) form a separate cluster from the non-overlapping 2R*d* genes (in orange). This could be explained in part by the covariance that is relatively tightly linked by virtue of physical proximity of any two genes.

Not all documented inversions will cause a clear clustering of genes in the t-SNE. Only inversions that are polymorphic in the samples analysed will have this effect.

### Centromere and telomere proximity

**Fig. 6:**
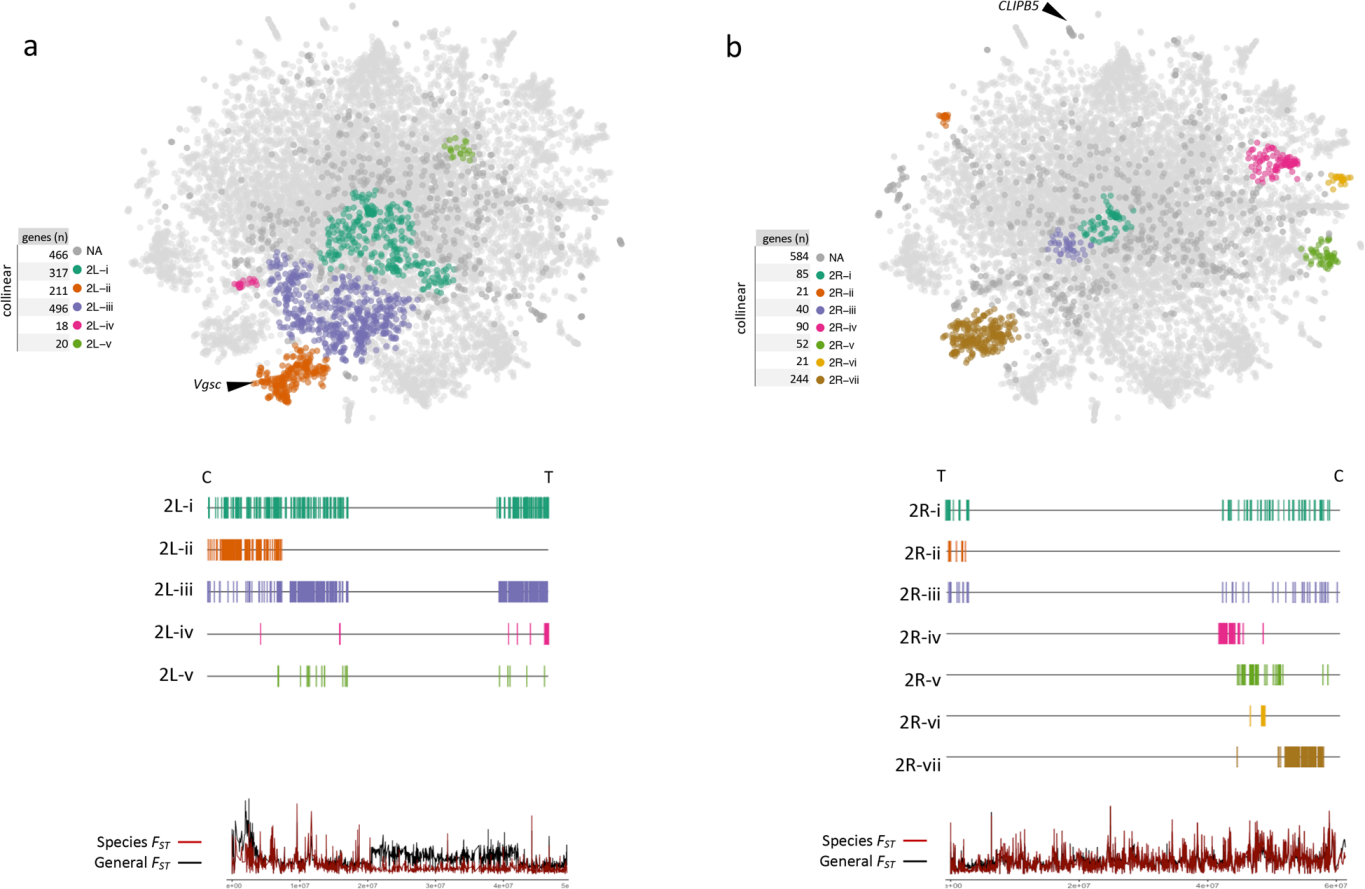
Cluster identification in chromosome 2. Automated cluster identification using the DBSCAN algorithm for genes in chromosome 2 outside the main known inversions. DBSCAN clusters are represented in color. Below each tSNE plot is a schematic representation of the chromosome and the starting points of each gene in a cluster is represented by a vertical line. Species *F*_*ST*_ computed between *A. gambiae* and *A. coluzzii* (red line), and general population *F*_*ST*_ (black line). “C” for centromere and “T” telomere. **a** Five clusters are found in the 2L chromosome (2L-i – 2L-v). **b** Seven clusters are found in the 2R chromosome (2Ri - 2Rvii). NA – gene not assigned to any cluster. Black arrows indicate key genes referenced in the text

**Fig. 7:**
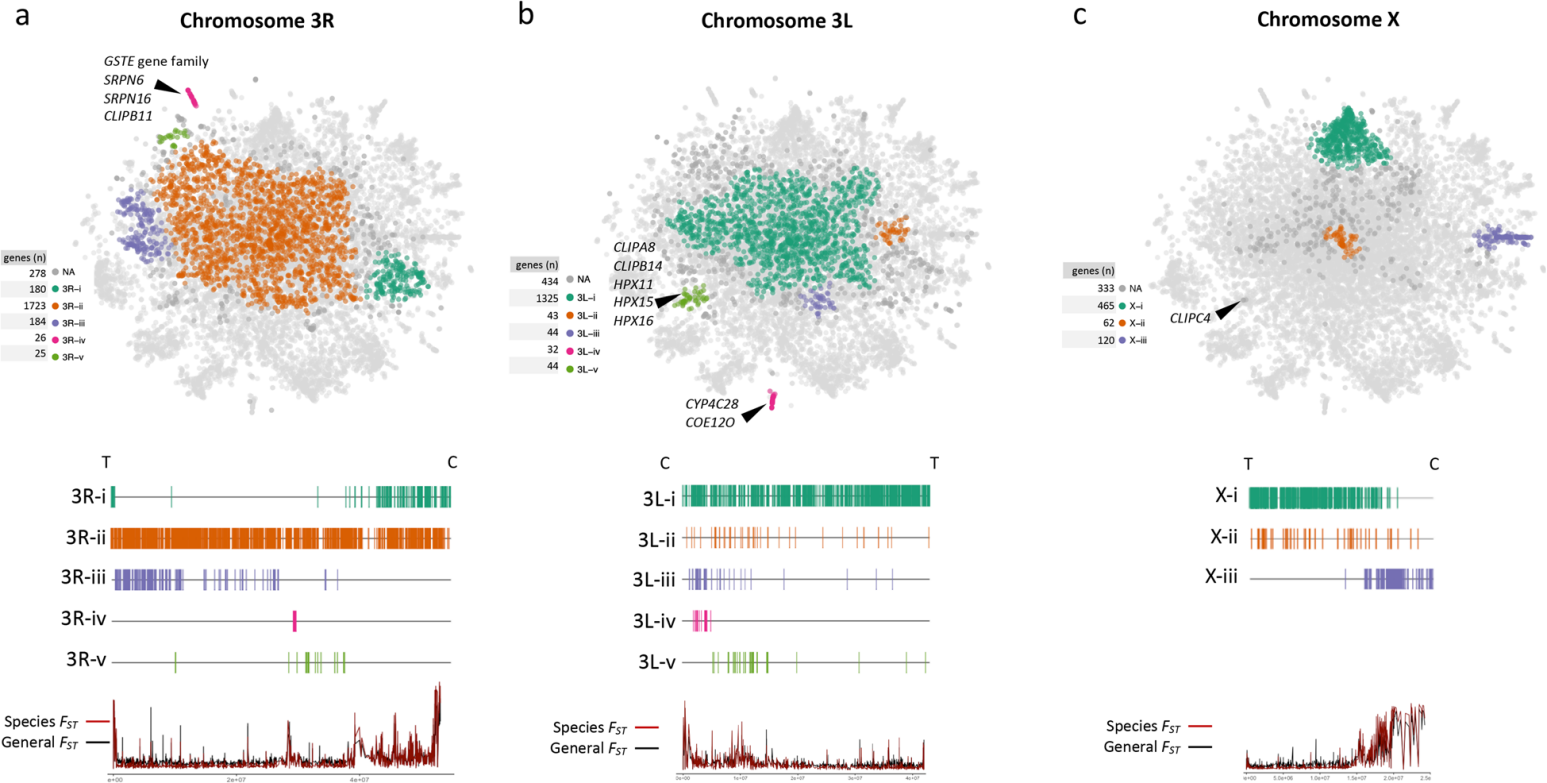
Cluster identification in chromosomes 3 and X. Automated DBSCAN cluster identification for non-inversion-located genes in chromosomes 3R **a**, 3L **b** and X **c**. See Fig. 6 for details. NA – gene not assigned to any cluster. Black arrows indicate key genes referenced in the text

Several well-defined peripheral clusters are formed from genes not located within inversions and are worthy of further investigation. We have used the algorithm DBScan to extract clusters for each chromosome arm outside of the main inversions (S7 Table). Two of the largest non-inversion clusters are 2R-vii and 2L-ii, each containing more than 200 genes that are located close to the centromere (Fig. 6). Cluster X-iii (120 genes, Fig. 7c), previously discussed in terms of sibling species differentiation, is also centromere-proximal. The distinctness of these clusters may be explained by lower rates of recombination around centromeres that has been shown in several animals, plants and fungi [53], since reduced mixing would make it more likely that two neighbouring genes share the same population structure.

Centromere-proximal genes on chromosome 3 do not form consistently large peripheral clusters analogous to clusters 2R-vii and 2L-ii. Cluster 3R-i (180 genes, Fig. 7a) is large and consistent, but is rarely truly peripheral like 2R-vii and 2L-ii. Centromere-proximal clusters on 3L are small and one (3L-iv) is discussed below in another context. How the centromeres of the two autosomes have come to have different population genetic dynamics remains to be explained, though the presence of chromosomal inversions in one chromosome but not the other may be a factor.

Genes close to telomeres are not locally constrained in the t-SNE plot to the degree seen for genes in inversions and centromeres regions. For example, on chromosome 2, only two small t-SNE clusters are found near the telomeres 2R-ii (21 genes, Fig. 6b) and 2L-iv (18 genes, Fig. 6a), and near the telomere of chromosome 3R, cluster 3R-iii is quite large (184 genes, Fig. 7a) though not clearly separated from the core region of t-SNE.

### Selective sweeps

Another genetic factor that strongly influences population structure and therefore the layout of genes in the t-SNE is positive selection. If a single locus is under strong selection, its genomic neighborhood is also affected due to linkage disequilibrium (LD), creating a so-called selective sweep. Positive selection is typically identified through the analysis of haplotype diversity and LD with reference to a fully assembled genome [54, 55]. In this study, we note that small, isolated clusters of contiguous genes in the t-SNE typically contain a gene that has either previously been implicated in recent selective sweeps (often related to insecticide resistance) or is a likely candidate for such selection. Thus, our t-SNE of gene-resolution population structure may offer a simple visual means to identify potential selective sweep genes in organisms with poorly assembled genomes. Below we explore in detail individual genes and genomic regions under selection.

Perhaps the most prominent ‘selective sweep cluster’ is the ‘GSTE cluster’, 3R-iv (Fig. 7a), containing four glutathione S-transferase genes: *GSTE1 (*AGAP009195), *GSTE5 (*AGAP009192*)*, *GSTE6 (*AGAP009191*)* and *GSTE7 (*AGAP009196*)* that exhibit high population structure (General *F*_*ST*_ respectively = 0.27, 0.25, 0.29 and 0.25; S6 Table). This region of strong selection was also identified in the original analysis of the Ag1000G dataset [22]. The genes *GSTE2 (*AGAP009194*)*, *GSTE3 (*AGAP009197*)* and *GSTE4 (*AGAP009193*)* are also located in this genomic region but fall in the center of the t-SNE due to low numbers of SNPs that pass the quality criteria. It is thought that *GSTE2* may be the actual gene under selection [56, 57]. Three immune system genes *SRPN6*, *SRPN16* and *CLIPB11* are also consistently present on this cluster, however it is not clear if these have evolutionary and functional significance or have simply piggybacked with the locus under selection. It is noteworthy that *SRPN6* is highly expressed in mosquito midgut and salivary gland epithelial cells that are invaded by the malaria parasites and is involved in parasite killing and/or clearance [58, 59]. Therefore, its putative involvement in this selective sweep notwithstanding, its location within a strongly selected locus could contribute to diversifying vectorial capacity between *An. gambiae* populations.

The tandemly duplicated *CYP6P* gene family has been previously identified to be under recent selection and likely involved in insecticide resistance [60–62]. In the t-SNE, the genes of this family are excluded because they are located in the intron of another gene, AGAP002859. However, this gene and 56 neighbouring genes form an isolated cluster (yellow cluster in Fig. 5b). All 57 genes are located within the 2R*c* inversion, though the majority of the 271 genes within this inversion are dispersed elsewhere on the t-SNE (Fig. 5b; web interface). Thus the ‘2R*c* cluster’ in Fig. 5b is not a typical inversion cluster as seen for 2L*a* or 2R*b* on the t-SNE plot (see Fig. 2), for instance, and may be better characterised as a selective sweep.

Both of the small, isolated, contiguous clusters 2L*a*-2 and 3L-iv contain cytochrome P450 genes. Cluster 2L*a*-2 (Fig. 5a) contains *CYP4J5* (AGAP006048), *CYP4J10* (AGAP006049) and *CYP4J9* (AGAP006047). Weetman et al. [20] identified SNPs in *CYP4J5* and *CYP4J10* that are associated with pyrethroid resistance in Ugandan isofemale families but only one of the SNPs in the *CYP4J5* gene showed highly reproducible and significant resistance association in sample sets from both Uganda and Kenya [20]. Because there was no loss of haplotypic diversity in the few samples sequenced from Uganda, they suggested that *CYP4J5* has been subject to a soft selective sweep. However, the consistent distinctness of the 2L*a*-2 cluster in our analysis of 765 samples suggests a strong selective sweep has indeed occurred. The 3L-iv cluster (Fig. 7b) is likely the result of selection on *CYP4C28* (AGAP010414) or carboxylesterase *COE12O* (AGAP010390). The former gene is overexpressed in mosquitoes collected from agricultural sites compared to an insecticide susceptible strain, suggesting involvement in insecticide resistance [63].

Another small, isolated cluster of 15 genes, containing the immunity-related gene *CLIPB5* (AGAP004148) may also indicate a recent selective sweep (Fig. 6b). Notably, this region (2R:50645302–50,862,651) does not contain any genes typically associated with insecticide resistance and so *CLIPB5* may be the most likely candidate to contain the allele under selection [64].

The best-known gene under strong selective pressure in insects is *Vgsc* (*para* gene - AGAP004707). Two mutations in *Vgsc* codon 995 of *An. gambiae* have conferred knockdown resistance (*kdr*) to DDT and pyrethroid insecticides: leucine to phenylalanine (L995F) [65] and leucine to serine (S995) [66]. The frequency of L995F *kdr* mutation is high in West and Central Africa populations included in the present study, while L995S is mostly present in Central and East Africa [22]. This gene is located in the centromere-proximal region of the left arm of chromosome 2 and it belongs to cluster 2L-ii in the t-SNE (Fig. 6a), a cluster much larger (200 genes) than the selective sweep clusters (20–30 genes) described above. Thus, visual interpretation of the t-SNE would not highlight this locus as a potential selective sweep. The complex multi-locus resistance of the *Vgsc* gene, its multiple introgressions between sibling species, and its location close to the centromere may explain why this gene does not belong to a small, isolated cluster typical of other recently selected genes.

### The Guinea-Bissau cluster

**Fig. 8:**
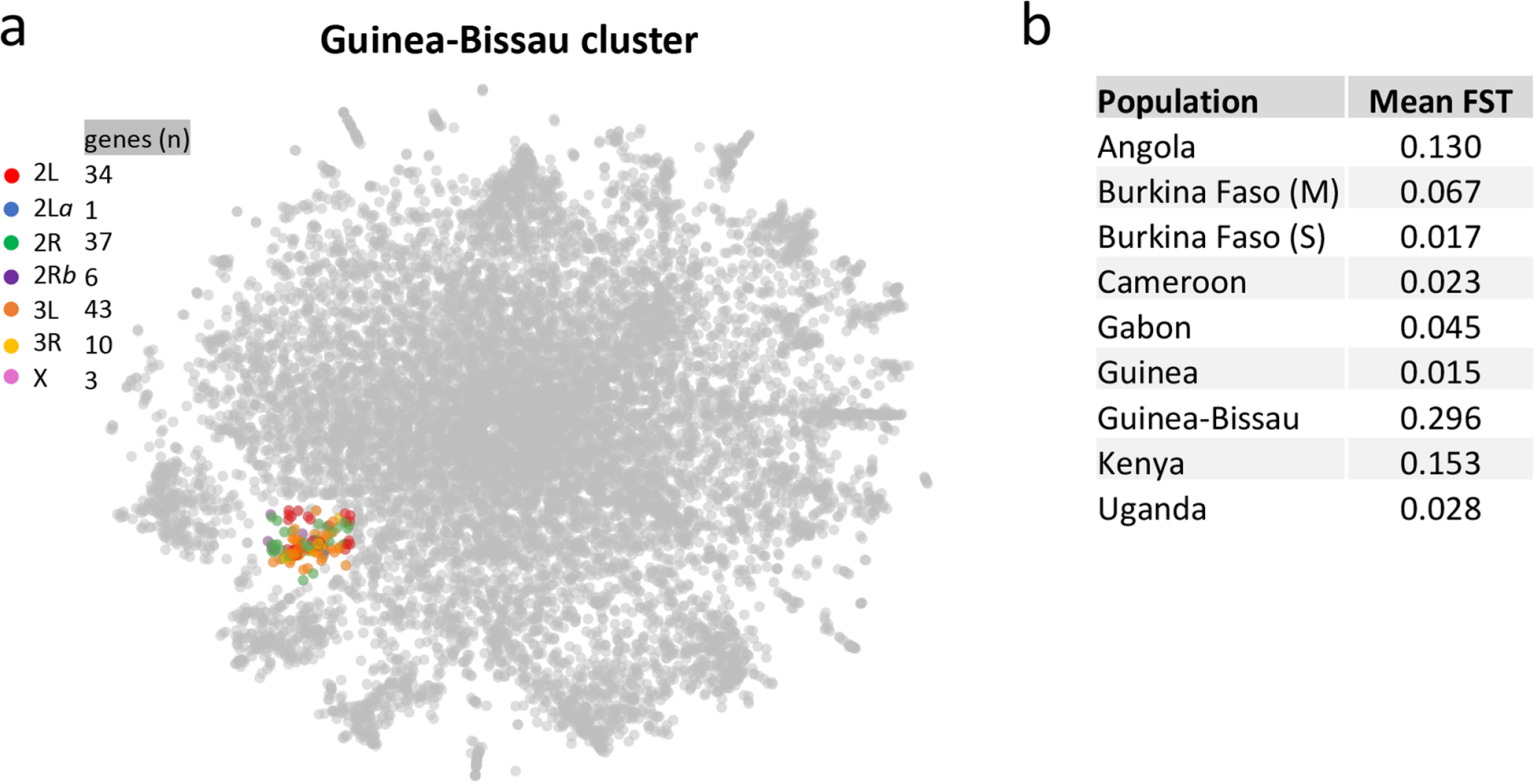
Guinea Bissau cluster. **a** t-SNE plot highlighting genes in the “Guinea Bissau cluster”, which are colored according to chromosome arm location and/or main known inversion in the *A. gambiae* genome. **b** Mean *Population F*_*ST*_ for genes within the cluster

It is also possible for selection to act on two or more unlinked loci. Clusters 2L-iv (Fig. 6a) and 3L-v (Fig. 7b) overlap in an area of the t-SNE that we henceforth refer to as the “Guinea-Bissau cluster” (Fig. 8) since genes within this cluster exhibit the highest *Population F*_*ST*_ for this country (S4 Fig; S8 Table). Guinea Bissau samples included in present study are from the coastal region, where introgression from *An. coluzzii* to *An. gambiae* has been persistently reported at high rates (> 20%) [25, 26, 65]. Vicente et al. [67] reported that this massive introgression, which is limited to the coastal region, drove species radiation between coastal and inland *An. gambiae* populations. The spatiotemporal stability of this novel hybrid form was related to species and local selection on chromosomal inversions. Whilst inland *An. gambiae* populations showed a common chromosomal form (SAVANNA) across West Africa, coastal *A. gambiae* presented a localized chromosomal form (BISSAU). The “Guinea-Bissau cluster” could reveal genes involved in local adaptation of this hybrid form.

The cluster contains just over 100 genes from both autosomes and three genes from the X chromosome. Several putative immunity-related genes are present including *CLIPC4* (AGAP000573), *CLIPA8* (AGAP010731), *CLIPB14* (AGAP010833), *HPX11* (AGAP010899), *HPX15* (AGAP013327) and *HPX16* (AGAP011216). The heme peroxidase (*HPX*) genes are of particular note being located so close together in the map despite not being close genomic neighbours (spanning a region of 300 genes), and because *HPX15* (also known as *IMPer*) has been implicated in the modulation of midgut immunity and microbiota tolerance [68].

Interestingly, two of the four genes in this cluster with the highest Guinea-Bissau *Population F*_*ST*_ have published links to viral infections: AGAP010732 (*F*_*ST*_ = 0.71) encodes a zinc-finger protein which is significantly upregulated upon densovirus infection (Ren et al., 2014); and AGAP004695 (*F*_*ST*_ = 0.75), which encodes a subunit of the ESCRT-I complex that mediates the intracellular trafficking of membrane proteins, was found to be upregulated during O’Nyong Nyong virus (ONNV) infection [69]. The gene encoding eukaryotic translation initiation factor 3 subunit B (AGAP012140) is also located in this cluster, though with a lower *F*_*ST*_ of 0.14, providing further support for a local viral challenge hypothesis given that viruses are dependent on the host’s translation machinery [70]. However, the innate immunity genes *CLIPA8* and *CLIPB14* that are also found in this cluster, are associated with *Plasmodium* and bacterial infections [71, 72], so non-viral immune challenges may also have influenced the evolution of these genes in Guinea-Bissau.

### Gene function enrichment in the t-SNE

A systematic analysis was performed to detect over-representation of gene functions in sub-regions of the t-SNE. *K*-means clustering using the 2D t-SNE coordinates was exhaustively performed for a variety of *K* values (see Methods for details) to partition the map into different subsets. Each gene set was tested for overrepresentation of biological function by means of a Gene Ontology (GO) term enrichment analysis using annotations from VectorBase [27]. After appropriate multiple testing corrections, 67 unique GO terms were found to be significantly enriched in various locations in the t-SNE (S6 Fig; S9 Table).

Since genomic location is the primary driver of the location of a gene in the t-SNE, tandemly duplicated genes are generally found close together in the plot and their GO terms are enriched, though only trivially. Therefore, we were particularly interested in GO terms enriched in clusters of non-contiguous genes. Broadly speaking, the center of the plot is characterized by a low number of SNPs as well as low population structure values, i.e. *General* and *Species F*_*ST*_ (Fig. 3), characteristic of conserved/housekeeping genes. As expected, GO terms related to basic maintenance of biological functions such as translation, peptide and amide biosynthesis, ribosome, mitochondria are enriched in that area (S6 Fig).

The t-SNE region described above as the “species divergence cluster” contains genes from different chromosome arms (Figs. 2; 4d). Interestingly, this area is enriched for sensory perception and behavior (S6 Fig; S9 Table), functions likely to be involved in the distinct mating and habitat preferences of *An. gambiae* and *An. coluzzii*. The “Guinea-Bissau cluster”, which, as discussed above, contains several highly differentiated genes putatively involved in viral infection, is also significantly enriched for cholesterol transport and ion binding. All four cholesterol transport genes are located in a tandem array within the 2R*c* inversion, so the GO enrichment is not unexpected. However, the majority of the genes closely neighbouring the tandem array are found in other distinct clusters in the t-SNE, particularly the main 2R*c* cluster. So, the cholesterol transport genes appear to be in the “Guinea-Bissau cluster” by exception rather than by default. Cholesterol transport and ion binding can be linked to viral infections: membrane lipid properties can affect viral entry and exit and intracellular trafficking, and the expression of ion binding genes was previously found altered in *Aedes aegypti* under flaviviral infection [73].

## Conclusion

Population genetic studies generally seek to make inferences about population structure, effective population size, evolutionary rates and incipient speciation, among others. Advances in DNA sequencing technologies have increased the depth and breadth of these studies, allowing long-standing biological questions to be addressed. Nonetheless, few studies explore population genetic variation from a genome-wide, gene-wise perspective. Here, we use t-SNE, a recently popularized dimensionality reduction method, to create a 2D-map of *An. gambiae* and *An. coluzzii* genes based on their population structure in 765 mosquitoes collected from across Africa that were previously sequenced and analyzed by the Ag1000G consortium. Our approach makes no assumptions about the division of individuals into sub-populations and provides a visualization of intra-genomic population structure differences that can relate to any subset of individuals under selection or reproductive isolation. The map allows intuitive navigation among genes distributed throughout the so-called “mainland” and numerous surrounding “island-like” clusters. These gene clusters of various sizes seem to a large extent to be driven by low recombination genomic regions such as inversions and proximity to centromeres, but also by recent selective sweeps. Because this mosquito species complex has been studied extensively, we were able to support our interpretations with previously published findings. Several novel observations have also been put forward here.

Looking forward, our methodology provides a powerful foundation to analyze and visualize population structure at gene-resolution in additional species, some of which are not so well characterized as *An. gambiae*, shortening the time required to progress from field sampling to the identification of genes and genomic regions under unique and biologically relevant evolutionary pressures. Unlike most population genetic analyses, this method does not require a fully assembled, “chromosome quality” reference genome, which is becoming a rarity in the current era of rapid sequencing of many species and individuals. In particular, our method can have applications in disease vector and pest control using means of genetic modification and gene drive, as it can rapidly identify chromosomal inversions and selective sweep regions that are likely to be poor targets for modification via gene drive technologies due to their atypical population dynamics. Big data visualisation and dimensionality-reducing embedding techniques continue to be developed. Successors to t-SNE, such as UMAP [74] and variational autoencoder neural networks [75] may further improve the visualization and interpretation of intra-genomic population structure heterogeneity.

## Methods

### Data preparation

All data were obtained from the *Anopheles gambiae* 1000 Genomes Project phase 1 (Ag1000G; https://www.malariagen.net/projects/ag1000). Mosquitoes were collected from natural populations at 15 locations in 8 African countries (S1 Table). Following alignment to the AgamP3 reference genome, variant calling was discovered and provided as per gene in Variant Call Format (VCF) files. For details, see Ag1000G [22].

In total, 11,318 VCF files for the gene files in chromosome arms 2R, 2L, 3R, 3L and X were analyzed (S2 Table). Exon-only SNPs were extracted using VCFtools [76] and missing data frequencies were verified and summarising individual-wise and SNP-wise for each gene. Each gene file was converted in Genomic Data Structure (GDS) using *SNPRelate* [77] package in R whereby each individual mosquito’s genotype at each SNP locus is summarised as a single integer to allow further analysis.

### Gene-wise population structure

Pairwise *F*_*ST*_ between the 9 defined populations was averaged (hereby referred to as *General F*_*ST*_) using *hierfstat* [78] package. This reflects the overall genetic population structure with respect to geography. Inter-group *F*_*ST*_ was also calculated using other available sample classifications, such as: species (*Species F*_*ST*_ between *An. coluzzii* and *An. gambiae s.s*), 2L*a* and 2R*b* karyotypes, and continental region (West, Central and East Africa) (S4 and S5 Figs; S5 Table). In addition, each population had per-gene *F*_*ST*_ calculated against all other populations combined, to indicate genes with unique evolutionary pressures for a particular geographic location. Intrapopulation genetic diversity was calculated using the same R package and available metadata.

### High-dimensionality reduction

The goal of dimensionality reduction is to preserve as much information of the high-dimensional data set in the low-dimensional representation. T-Distributed Stochastic Neighbor Embedding (t-SNE) was used to reduce n-dimensional information to two dimensions, where n is the linearized distance matrix per gene i.e. 292,230 dimensions. This technique is a non-linear algorithm, which preserves local structures while attempting to maintain global relationships.

Firstly, inter-mosquito distance matrices (765 × 765) were calculated for each gene using their vectors of GDS numbers by the Manhattan method using the *dist* package in R (Fig. 1). Then, linearized per-gene distance matrices were concatenated together. This large matrix (> 3 billion elements) was handled in R by using the *bigmemory* v.4 package. Initially, a PCA was performed with *bigpca* v.1 and the first 50 principal components were then used as input for the t-SNE runs in *Rtsne* [79]. Each point in the t-SNE plot then represents one gene and genes close together in the plot indicate population structure similarity. The parameters for t-SNE were theta 0, perplexity 500, number of iterations 5000. The perplexity parameter controls the size (in number of genes) of the local neighbourhood in high-dimensional space that the algorithm considers. Low perplexities can artificially strand data points in clusters that should really be connected. Some alternative parameter settings for perplexity are presented in Figure S7 and the web interface, where it can be seen that the overall arrangement and grouping of genes is not fundamentally changed.

DBScan is a density-based spatial algorithm used to find clusters of genes in the t-SNE [80] in R. This method requires choosing the maximum distance between data points (eps = 0.6) and minimum number to form a cluster (minPts = 15).

### Inter-t-SNE consistency

To verify the consistency of the t-SNE dimensionality reduction, which has a random initiation step, 30 independently seeded runs were performed. Two gene-wise metrics were calculated to summarise the variability of gene-gene spatial relationships within the 2D t-SNE mapping. The first measure, *mean_variance* is more globally motivated: first the variance of the distance between gene *i* and *j* over the 30 maps is calculated, then *mean_variance* for gene *i* is simply the mean of these variances for the distances from gene *i* to all genes *j ≠ i*. The second measure encapsulates local neighbour relationships: *number_unique_nearest_N_neighbours* is the total number of different genes seen as nearest *N* neighbours to gene *i* across the 30 maps. Its minimum is *N*, indicating that a gene always has the same *N* nearest neighbours regardless of the t-SNE initialization step, and its hypothetical maximum is 30 *N* indicating no consistency of local neighbourhood at all. Both measures (with the latter using *N* = 5,20,50) are presented in the interactive web interface, available at https://vigilab.shinyapps.io/anopheles/.

### Gene function over-representation analysis

The genes in the t-SNE were partitioned using *K*-means clustering on the 2D plot coordinates at different levels of granularity. Partitions were made with *K* = 2 to *K* = 30 and then in increments of 5 up to 100 (i.e. 35, 45, 50…). In total, 1409 gene sets were produced. Gene Ontology (GO) over-representation analysis using the *topGO* R package v.2.24.0 was performed on each cluster using a weighted Fisher’s Exact Test where the null hypothesis states that genes with a particular GO term are randomly distributed between the *k*-means clusters. The weighting procedure takes into account the hierarchical relationships between GO terms and, in effect, obviates the need for multiple testing correction with respect to the many GO terms analysed. An additional Bonferroni-like correction for the multiple *K*-means clusters tested was applied, resulting in a final *p*-value threshold of 10^− 5^.

## Supplementary Information

**Additional file 1: S1 Fig.** Replicated t-SNE plots. A total of 30 replicate t-SNE plots similar to Fig. 1 were produced using different random seeds. See Fig. 1 for details. The reproducible representation of specific gene sets can be explored using the web interface at https://vigilab.shinyapps.io/anopheles/.

**Additional file 2: S2 Fig.** Local consistency measures. Ranked distribution of the number of unique nearest neighbors for each gene. Three levels were used for nearest neighbours (5, 20 and 50) in order to measure consistency in small and large clusters. Genes with low values reflect higher consistency of the local neighborhood in the t-SNE plot. **S3 Fig.** Global consistency measures. Ranked distribution of the mean and median values of t-SNE coordinates distances between each gene and all other genes. Genes with low values reflect higher consistency of global arrangement in the t-SNE plot. **S4 Fig.** Ranked distribution of individual country *F*_*ST*_ values. Population *F*_*ST*_ values calculated for each country/population vs. all other individuals. **S5 Fig.** Ranked distribution of broad geographic regions *F*_*ST*_ values. *F*_*ST*_ values between grouped countries in East, Central and West Africa. **S6 Fig.** Representation of the most significant gene function over-representation clusters. Coloured lines define the clusters within which a GO term was found enriched. The three GO categories: biological process, molecular function and cellular component, are analysed separately. **S7 Fig.** Additional t-SNE plots for varied perplexity values. Scatter plots of t-SNE using perplexity equals 50, 100, 250 and 1000.

**Additional file 3: S1 Table.** Sample information, data from the Ag1000G.

**Additional file 4: S2 Table.** Number of SNPs, genomic location and coordinates for each gene in the t-SNE plot.

**Additional file 5: S3 Table.** Summary of missing data per gene.

**Additional file 6: S4 Table.** Measure of consistency of the t-SNE plot.

**Additional file 7: S5 Table.** Fst values for each gene in the t-SNE plot.

**Additional file 8: S6 Table.** List of genes showing Fst > 0.3, 0.4, 0.5 between *An. gambiae* and *An. coluzzii*.

**Additional file 9: S7 Table.** Summary and list of genes within the DBScan cluster for each chromosome arm.

**Additional file 10: S8 Table.** Guinea-Bissau cluster.

**Additional file 11: S9 Table.** GO enrichment analysis.

## Data Availability

The interactive map is available at https://vigilab.shinyapps.io/anopheles/. Source code for the analysis and web interface is available athttps://github.com/melcampos/genewise-tSNE. Accession numbers for all samples on Table S1 are under study accession PRJEB18691 from the European Nucleotide Archive (ENA - http://www.ebi.ac.uk/ena). Data from Ag1000G phase 1 is available from the Ag1000G public FTP site via MalariaGEN website https://www.malariagen.net/data/ag1000g-phase1-ar3.1. Genomic sequences and gene annotations are available at https://vectorbase.org/. The AgamP3 genome assembly is available at:https://vectorbase.org/common/downloads/Legacy%20VectorBase%20Files/Anopheles-gambiae/Anopheles-gambiae-PEST_CHROMOSOMES_AgamP3.fa.gz

## Abbreviations

Ag1000G: *Anopheles gambiae* 1000 Genomes Consortium
DBScan: Density-based spatial algorithm
F_ST_: Fixation index
GDS: Genomic Data Structure
GO: Gene Ontology
IGS: Intergenic spacer
LD: Linkage disequilibrium
OBP: Odorant binding protein
ONNV: O’Nyong Nyong virus
PCA: Principal Component Analysis
SNP: Single Nucleotide Polymorphism
s.l.: sensu lato
s.s.: sensu stricto
t-SNE: t-Distributed Stochastic Neighbor Embedding
VCF: Variant Call Format

## References

[1] White GB (1974). Biological effects of intraspecific chromosomal polymorphism in malaria vector populations. Bull World Health Org.

[2] Coluzzi M, Sabatini A, della Torre A, Di Deco MA, Petrarca V (2002). A Polytene chromosome analysis of the *Anopheles gambiae* species complex. Science.

[3] Davidson G, Jackson CE (1962). Incipient speciation in Anopheles gambiae Giles. Bull World Health Organ.

[4] Coetzee M, Hunt RH, Wilkerson R, Torre AD, Coulibaly MB, Besansky NJ (2013). Anopheles coluzzii and Anopheles amharicus, new members of the Anopheles gambiae complex. Zootaxa..

[5] Coluzzi M, Sabatini A, Petrarca V, Di Deco MA (1979). Chromosomal differentiation and adaptation to human environments in the Anopheles gambiae complex. Trans R Soc Trop Med Hyg.

[6] Touré YT, Petrarca V, Traoré SF, Coulibaly A, Maiga HM, Sankaré O (1994). Ecological genetic studies in the chromosomal form Mopti of Anopheles gambiae s.str. in Mali, west Africa. Genetica.

[7] della Torre A, Fanello C, Akogbeto M, Dossou-yovo J, Favia G, Petrarca V (2001). Molecular evidence of incipient speciation within *Anopheles gambiae* s.s. in West Africa. Insect Mol Biol.

[8] Cheng C, Tan JC, Hahn MW, Besansky NJ (2018). Systems genetic analysis of inversion polymorphisms in the malaria mosquito Anopheles gambiae. Proc Natl Acad Sci U S A.

[9] Coluzzi M, Petrarca V, Di Deco MA (1985). Chromosomal inversion intergradation and incipient speciation in *Anopheles gambiae*. Italian J Zool.

[10] Kirkpatrick M, Barton N (2006). Chromosome inversions, local adaptation and speciation. Genetics..

[11] Powell JR, Petrarca V, della Torre A, Caccone A, Coluzzi M (1999). Population structure, speciation, and introgression in the *Anopheles gambiae* complex. Parassitologia.

[12] Simard F, Ayala D, Kamdem GC, Pombi M, Etouna J, Ose K, Fotsing JM, Fontenille D, Besansky NJ, Costantini C (2009). Ecological niche partitioning between Anopheles gambiae molecular forms in Cameroon: the ecological side of speciation. BMC Ecol.

[13] Fouet C, Gray E, Besansky NJ, Costantini C (2012). Adaptation to aridity in the malaria mosquito Anopheles gambiae: chromosomal inversion polymorphism and body size influence resistance to desiccation. PLoS One.

[14] Rocca KA, Gray EM, Costantini C, Besansky NJ (2009). 2La chromosomal inversion enhances thermal tolerance of Anopheles gambiae larvae. Malar J.

[15] Holt RA, Subramanian GM, Halpern A, Sutton GG, Charlab R, Nusskern DR (2002). The Genome Sequence of the Malaria Mosquito *Anopheles gambiae*. Science.

[16] White BJ, Cheng C, Simard F, Costantini C, Besansky NJ (2010). Genetic association of physically unlinked islands of genomic divergence in incipient species of Anopheles gambiae. Mol Ecol.

[17] Turner TL, Hahn MW, Nuzhdin SV (2005). Genomic islands of speciation in Anopheles gambiae. PLoS Biol.

[18] Lawniczak MK, Emrich SJ, Holloway AK, Regier AP, Olson M, White B (2010). Widespread divergence between incipient Anopheles gambiae species revealed by whole genome sequences. Science..

[19] Caputo B, Pichler V, Mancini E, Pombi M, Vicente JL, Dinis J, Steen K, Petrarca V, Rodrigues A, Pinto J, della Torre A, Weetman D (2016). The last bastion? X chromosome genotyping of Anopheles gambiae species pair males from a hybrid zone reveals complex recombination within the major candidate ‘genomic island of speciation’. Mol Ecol.

[20] Weetman D, Wilding CS, Neafsey DE, Muller P, Ochomo E, Isaacs AT (2018). Candidate-gene based GWAS identifies reproducible DNA markers for metabolic pyrethroid resistance from standing genetic variation in east African Anopheles gambiae. Sci Rep.

[21] Weetman D, Wilding CS, Steen K, Morgan JC, Simard F, Donnelly MJ (2010). Association mapping of insecticide resistance in wild Anopheles gambiae populations: major variants identified in a low-linkage disequilibrium genome. PLoS One.

[22] The Anopheles gambiae 1000 Genomes Consortium (2017). Genetic diversity of the African malaria vector *Anopheles gambiae*. Nature.

[23] Maaten L, Hinton G (2008). Visualizing Data using t-SNE. J Mach Learn Res.

[24] Maccallum RM, Redmond SN, Christophides GK (2011). An expression map for Anopheles gambiae. BMC Genomics.

[25] Marsden CD, Lee Y, Nieman CC, Sanford MR, Dinis J, Martins C (2011). Asymmetric introgression between the M and S forms of the malaria vector, Anopheles gambiae, maintains divergence despite extensive hybridization. Mol Ecol.

[26] Oliveira E, Salgueiro P, Palsson K, Vicente JL, Arez AP, Jaenson TG, Caccone A, Pinto J (2008). High levels of hybridization between molecular forms of Anopheles gambiae from Guinea Bissau. J Med Entomol.

[27] Giraldo-Calderon GI, Emrich SJ, MacCallum RM, Maslen G, Dialynas E, Topalis P (2015). VectorBase: an updated bioinformatics resource for invertebrate vectors and other organisms related with human diseases. Nucleic Acids Res.

[28] Jombart T, Kendall M, Almagro-Garcia J, Colijn C (2017). Treespace: statistical exploration of landscapes of phylogenetic trees. Mol Ecol Resour.

[29] Tzeng J, Lu HH, Li WH (2008). Multidimensional scaling for large genomic data sets. BMC Bioinformatics.

[30] Lee Y, Collier TC, Sanford MR, Marsden CD, Fofana A, Cornel AJ, Lanzaro GC (2013). Chromosome inversions, genomic differentiation and speciation in the African malaria mosquito Anopheles gambiae. PLoS One.

[31] Lee Y, Marsden CD, Norris LC, Collier TC, Main BJ, Fofana A, Cornel AJ, Lanzaro GC (2013). Spatiotemporal dynamics of gene flow and hybrid fitness between the M and S forms of the malaria mosquito, Anopheles gambiae. Proc Natl Acad Sci U S A.

[32] Pombi M, Kengne P, Gimonneau G, Tene-Fossog B, Ayala D, Kamdem C, Santolamazza F, Guelbeogo WM, Sagnon N’F, Petrarca V, Fontenille D, Besansky NJ, Antonio-Nkondjio C, Dabiré RK, della Torre A, Simard F, Costantini C (2017). Dissecting functional components of reproductive isolation among closely related sympatric species of the Anopheles gambiae complex. Evol Appl.

[33] Clarkson CS, Weetman D, Essandoh J, Yawson AE, Maslen G, Manske M, Field SG, Webster M, Antão T, MacInnis B, Kwiatkowski D, Donnelly MJ (2014). Adaptive introgression between Anopheles sibling species eliminates a major genomic island but not reproductive isolation. Nat Commun.

[34] Norris LC, Main BJ, Lee Y, Collier TC, Fofana A, Cornel AJ, Lanzaro GC (2015). Adaptive introgression in an African malaria mosquito coincident with the increased usage of insecticide-treated bed nets. Proc Natl Acad Sci U S A.

[35] Besansky NJ, Krzywinski J, Lehmann T, Simard F, Kern M, Mukabayire O, Fontenille D, Toure Y, Sagnon N (2003). Semipermeable species boundaries between Anopheles gambiae and Anopheles arabiensis: evidence from multilocus DNA sequence variation. Proc Natl Acad Sci U S A.

[36] Fontaine MC, Pease JB, Steele A, Waterhouse RM, Neafsey DE, Sharakhov IV (2015). Mosquito genomics. Extensive introgression in a malaria vector species complex revealed by phylogenomics. Science.

[37] Aboagye-Antwi F, Alhafez N, Weedall GD, Brothwood J, Kandola S, Paton D, Fofana A, Olohan L, Betancourth MP, Ekechukwu NE, Baeshen R, Traorè SF, Diabate A, Tripet F (2015). Experimental swap of Anopheles gambiae’s assortative mating preferences demonstrates key role of X-chromosome divergence island in incipient sympatric speciation. PLoS Genet.

[38] Lee Y, Marsden CD, Nieman C, Lanzaro GC (2014). A new multiplex SNP genotyping assay for detecting hybridization and introgression between the M and S molecular forms of Anopheles gambiae. Mol Ecol Resour.

[39] Neafsey DE, Lawniczak MKN, Park DJ, Redmond SN, Coulibaly MB, Traore SF, Sagnon N, Costantini C, Johnson C, Wiegand RC, Collins FH, Lander ES, Wirth DF, Kafatos FC, Besansky NJ, Christophides GK, Muskavitch MAT (2010). SNP genotyping defines complex gene-flow boundaries among African malaria vector mosquitoes. Science..

[40] Reidenbach KR, Neafsey DE, Costantini C, Sagnon N, Simard F, Ragland GJ (2012). Patterns of genomic differentiation between ecologically differentiated M and S forms of Anopheles gambiae in west and Central Africa. Genome Biol Evol.

[41] Fanello C, Santolamazza F, della Torre A (2002). Simultaneous identification of species and molecular forms of the Anopheles gambiae complex by PCR-RFLP. Med Vet Entomol.

[42] Scott JA, Brogdon WG, Collins FH (1993). Identification of single specimens of the Anopheles gambiae complex by the polymerase chain reaction. Am J Trop Med Hyg.

[43] Santolamazza F, Mancini E, Simard F, Qi Y, Tu Z, della Torre A (2008). Insertion polymorphisms of SINE200 retrotransposons within speciation islands of Anopheles gambiae molecular forms. Malar J.

[44] Baker DA, Nolan T, Fischer B, Pinder A, Crisanti A, Russell S (2011). A comprehensive gene expression atlas of sex- and tissue-specificity in the malaria vector, *Anopheles gambiae*. BMC Genomics.

[45] Marinotti O, Calvo E, Nguyen QK, Dissanayake S, Ribeiro JM, James AA (2006). Genome-wide analysis of gene expression in adult *Anopheles gambiae*. Insect Mol Biol.

[46] Amenya DA, Chou W, Li J, Yan G, Gershon PD, James AA (2010). Proteomics reveals novel components of the *Anopheles gambiae* eggshell. J Insect Physiol.

[47] Papa F, Windbichler N, Waterhouse RM, Cagnetti A, d’Amato R, Persampieri T (2017). Rapid evolution of female-biased genes among four species of Anopheles malaria mosquitoes. Genome Res.

[48] White BJ, Lawniczak MK, Cheng C, Coulibaly MB, Wilson MD, Sagnon N (2011). Adaptive divergence between incipient species of Anopheles gambiae increases resistance to Plasmodium. Proc Natl Acad Sci U S A.

[49] Markianos K, Bischoff E, Mitri C, Guelbeogo WM, Gneme A, Eiglmeier K, Holm I, Sagnon N’F, Vernick KD, Riehle MM (2016). Genetic structure of a local population of the Anopheles gambiae complex in Burkina Faso. PLoS One.

[50] Hanemaaijer MJ, Higgins H, Eralp I, Yamasaki Y, Becker N, Kirstein OD, Lanzaro GC, Lee Y (2019). Introgression between Anopheles gambiae and Anopheles coluzzii in Burkina Faso and its associations with kdr resistance and Plasmodium infection. Malar J.

[51] Kirkpatrick M (2010). How and why chromosome inversions evolve. PLoS Biol.

[52] Cheng C, White BJ, Kamdem C, Mockaitis K, Costantini C, Hahn MW, Besansky NJ (2012). Ecological genomics of Anopheles gambiae along a latitudinal cline: a population-resequencing approach. Genetics..

[53] Talbert PB, Henikoff S (2010). Centromeres convert but don’t cross. PLoS Biol.

[54] Chen H, Patterson N, Reich D (2010). Population differentiation as a test for selective sweeps. Genome Res.

[55] Sabeti PC, Reich DE, Higgins H, Levine HZ, Richter DJ, Schaffner SF (2002). Detecting recent positive selection in the human genome from haplotype structure. Nature.

[56] Riveron JM, Yunta C, Ibrahim SS, Djouaka R, Irving H, Menze BD, Ismail HM, Hemingway J, Ranson H, Albert A, Wondji CS (2014). A single mutation in the GSTe2 gene allows tracking of metabolically based insecticide resistance in a major malaria vector. Genome Biol.

[57] Mitchell SN, Rigden DJ, Dowd AJ, Lu F, Wilding CS, Weetman D, Dadzie S, Jenkins AM, Regna K, Boko P, Djogbenou L, Muskavitch MAT, Ranson H, Paine MJI, Mayans O, Donnelly MJ (2014). Metabolic and target-site mechanisms combine to confer strong DDT resistance in Anopheles gambiae. PLoS One.

[58] Abraham EG, Pinto SB, Ghosh A, Vanlandingham DL, Budd A, Higgs S, Kafatos FC, Jacobs-Lorena M, Michel K (2005). An immune-responsive serpin, SRPN6, mediates mosquito defense against malaria parasites. PNAS..

[59] Pinto SB, Kafatos FC, Michel K (2008). The parasite invasion marker SRPN6 reduces sporozoite numbers in salivary glands of Anopheles gambiae. Cell Microbiol.

[60] Weedall GD, Mugenzi LMJ, Menze BD, Tchouakui M, Ibrahim SS, Amvongo-Adjia N, Irving H, Wondji MJ, Tchoupo M, Djouaka R, Riveron JM, Wondji CS (2019). A cytochrome P450 allele confers pyrethroid resistance on a major African malaria vector, reducing insecticide-treated bednet efficacy. Sci Transl Med.

[61] Muller P, Warr E, Stevenson BJ, Pignatelli PM, Morgan JC, Steven A (2008). Field-caught permethrin-resistant Anopheles gambiae overexpress CYP6P3, a P450 that metabolises pyrethroids. PLoS Genet.

[62] Edi CV, Djogbenou L, Jenkins AM, Regna K, Muskavitch MA, Poupardin R (2014). CYP6 P450 enzymes and ACE-1 duplication produce extreme and multiple insecticide resistance in the malaria mosquito Anopheles gambiae. PLoS Genet.

[63] Nkya TE, Akhouayri I, Poupardin R, Batengana B, Mosha F, Magesa S, Kisinza W, David JP (2014). Insecticide resistance mechanisms associated with different environments in the malaria vector Anopheles gambiae: a case study in Tanzania. Malar J.

[64] Volz J, Müller H, Zdanowicz A, Kafatos FC, Osta MA (2006). A genetic module regulates the melanization response of Anopheles to Plasmodium. Cell Microbiol.

[65] Martinez-Torres D, Chandre F, Williamson MS, Darriet F, Bergé JB, Devonshire AL (1998). Molecular characterization of pyrethroid knockdown resistance (kdr) in the major malaria vector *Anopheles gambiae* s.s. Insect Mol Biol.

[66] Ranson H, N’Guessan R, Lines J, Moiroux N, Nkuni Z, Corbel V (2011). Pyrethroid resistance in African anopheline mosquitoes: what are the implications for malaria control?. Trends Parasitol.

[67] Vicente JL, Clarkson CS, Caputo B, Gomes B, Pombi M, Sousa CA, Antao T, Dinis J, Bottà G, Mancini E, Petrarca V, Mead D, Drury E, Stalker J, Miles A, Kwiatkowski DP, Donnelly MJ, Rodrigues A, Torre A, Weetman D, Pinto J (2017). Massive introgression drives species radiation at the range limit of Anopheles gambiae. Sci Rep.

[68] Kumar S, Molina-Cruz A, Gupta L, Rodrigues J, Barillas-Mury C (2010). A peroxidase/dual oxidase system modulates midgut epithelial immunity in Anopheles gambiae. Science..

[69] Waldock J, Olson KE, Christophides GK (2012). Anopheles gambiae antiviral immune response to systemic O'nyong-nyong infection. PLoS Negl Trop Dis.

[70] Bushell M, Sarnow P (2002). Hijacking the translation apparatus by RNA viruses. J Cell Biol.

[71] Volz J, Osta MA, Kafatos FC, Muller HM (2005). The roles of two clip domain serine proteases in innate immune responses of the malaria vector Anopheles gambiae. J Biol Chem.

[72] Yassine H, Kamareddine L, Osta MA (2012). The mosquito melanization response is implicated in defense against the entomopathogenic fungus Beauveria bassiana. PLoS Pathog.

[73] Colpitts TM, Cox J, Vanlandingham DL, Feitosa FM, Cheng G, Kurscheid S, Wang P, Krishnan MN, Higgs S, Fikrig E (2011). Alterations in the Aedes aegypti transcriptome during infection with West Nile, dengue and yellow fever viruses. PLoS Pathog.

[74] McInnes L, Healy J, Melville J (2018). UMAP: Uniform manifold approximation and projection for dimension reduction. arxiv.

[75] Battey CJ, Coffing GC, Kern AD (2021). Visualizing population structure with variational autoencoders. G3 (Bethesda).

[76] Danecek P, Auton A, Abecasis G, Albers CA, Banks E, DePristo MA (2011). The variant call format and VCFtools. Bioinformatics..

[77] Zheng X, Levine D, Shen J, Gogarten SM, Laurie C, Weir BS (2012). A high-performance computing toolset for relatedness and principal component analysis of SNP data. Bioinformatics..

[78] Goudet J (2005). Hierfstat, a package for R to compute and test variance components and F-statistics. Mol Ecol Notes.

[79] Maaten L (2014). Accelerating t-SNE using tree-based algorithms. J Mach Learn Res.

[80] Ester M, Kriegel H, Sander J, Xu X (1996). A density-based algorithm for discovering clusters in large spatial databases with noise. Proceedings.

