# Supplementary material for: Unravelling population structure heterogeneity within the genome of the malaria vector Anopheles gambiae
Source: BMC Genomics. 2021 Jun 8;22:422. doi: 10.1186/s12864-021-07722-y (PMC8185951; doi:10.1186/s12864-021-07722-y)

tSNE plot 01

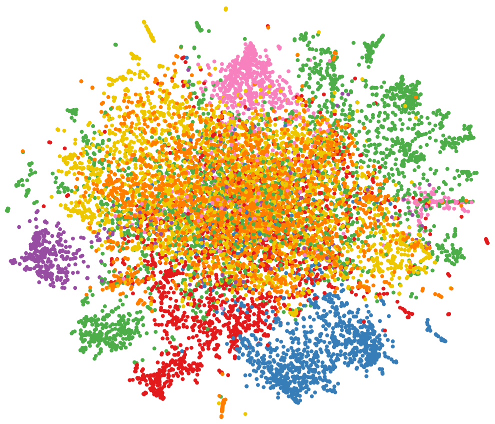

tSNE plot 02

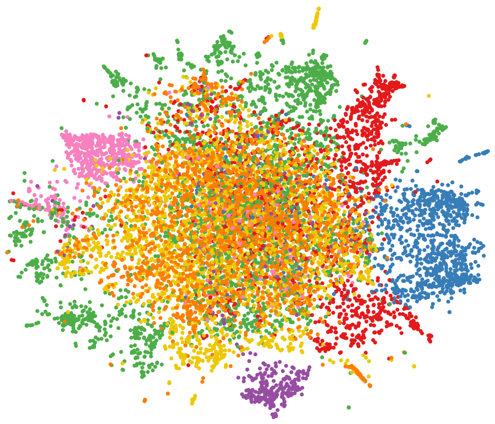

tSNE plot 03

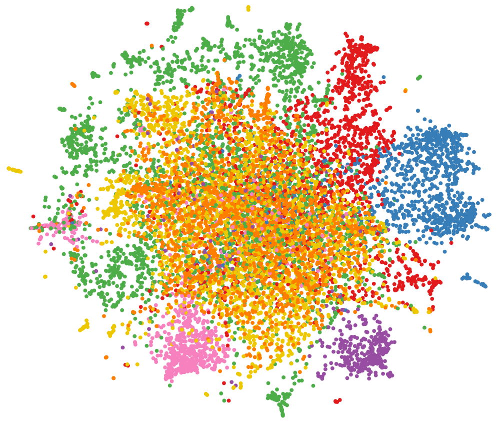

tSNE plot 04

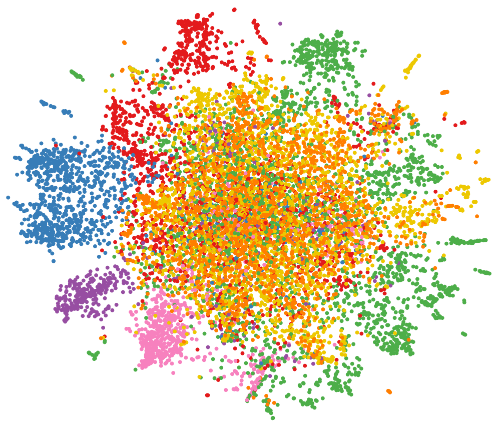

tSNE plot 05

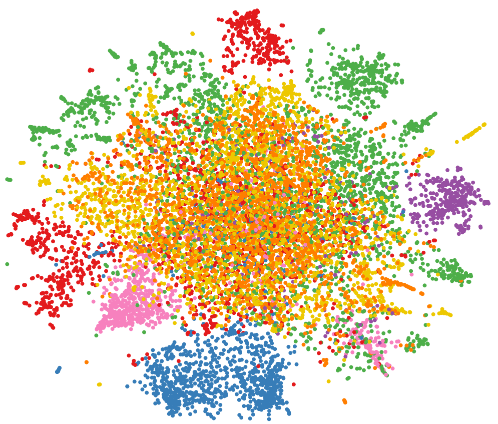

tSNE plot 06

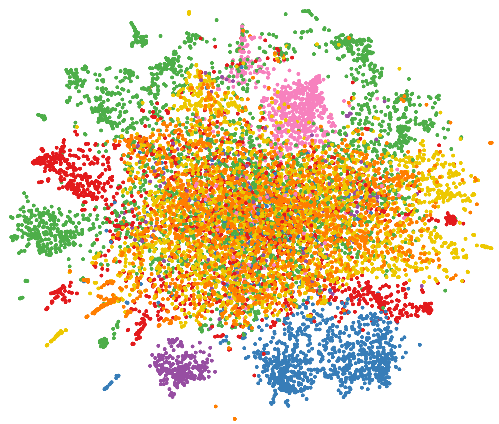

tSNE plot 07

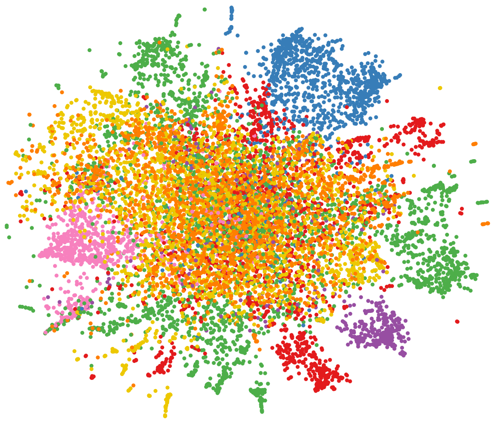

tSNE plot 08

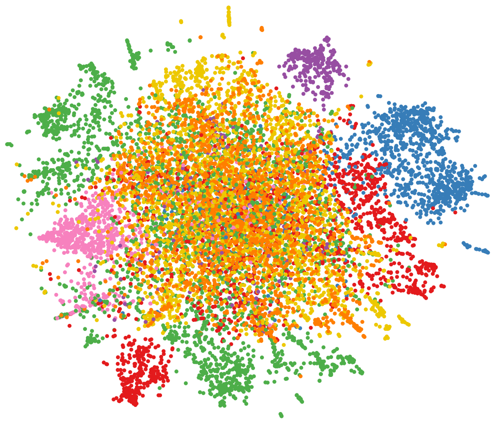

tSNE plot 09

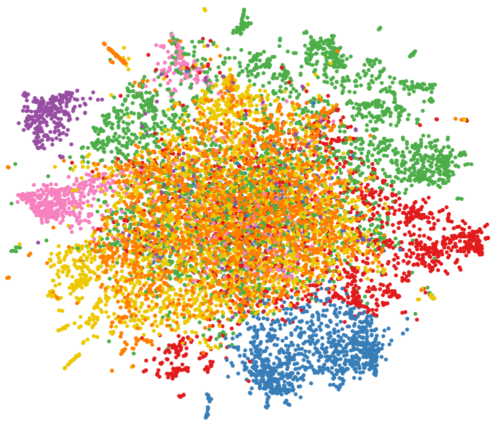

tSNE plot 10

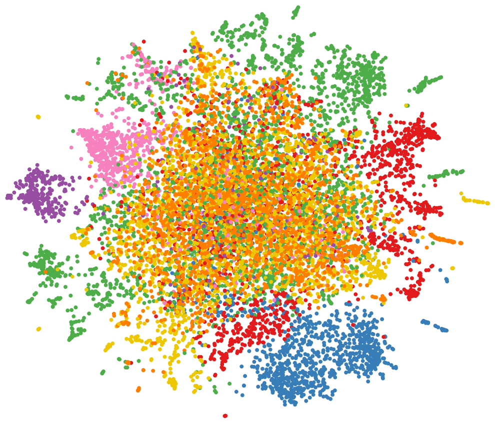

tSNE plot 11

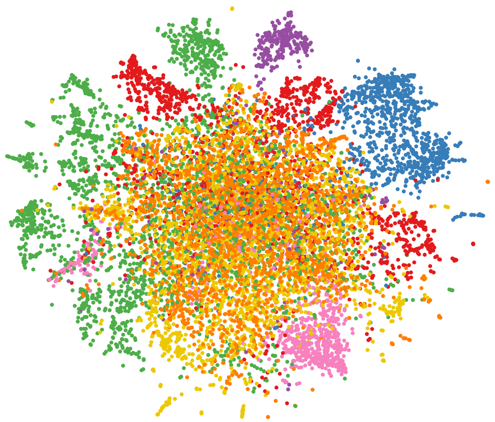

tSNE plot 12

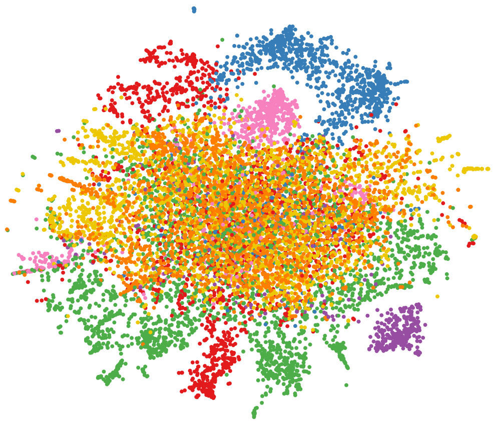

tSNE plot 13

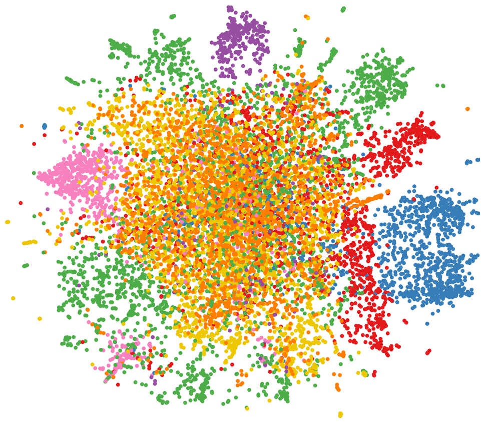

tSNE plot 14

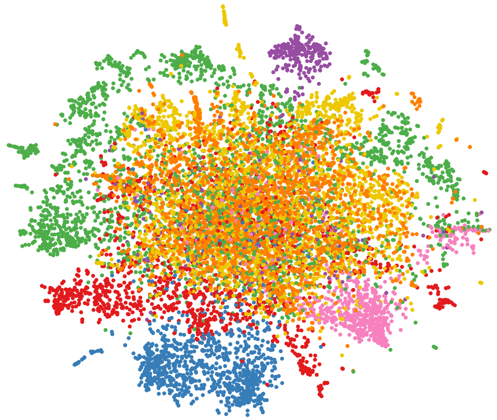

tSNE plot 15

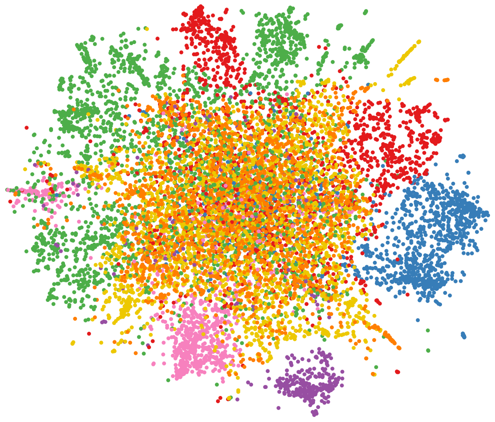

tSNE plot 16

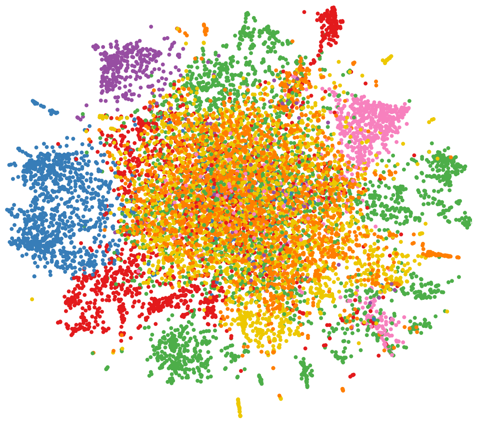

tSNE plot 17

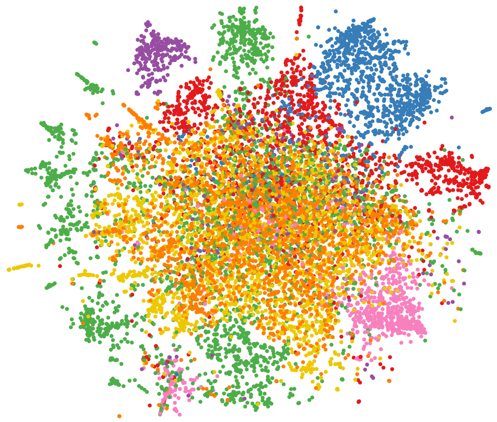

tSNE plot 18

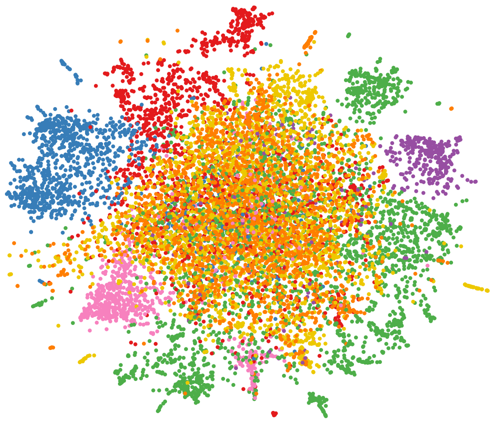

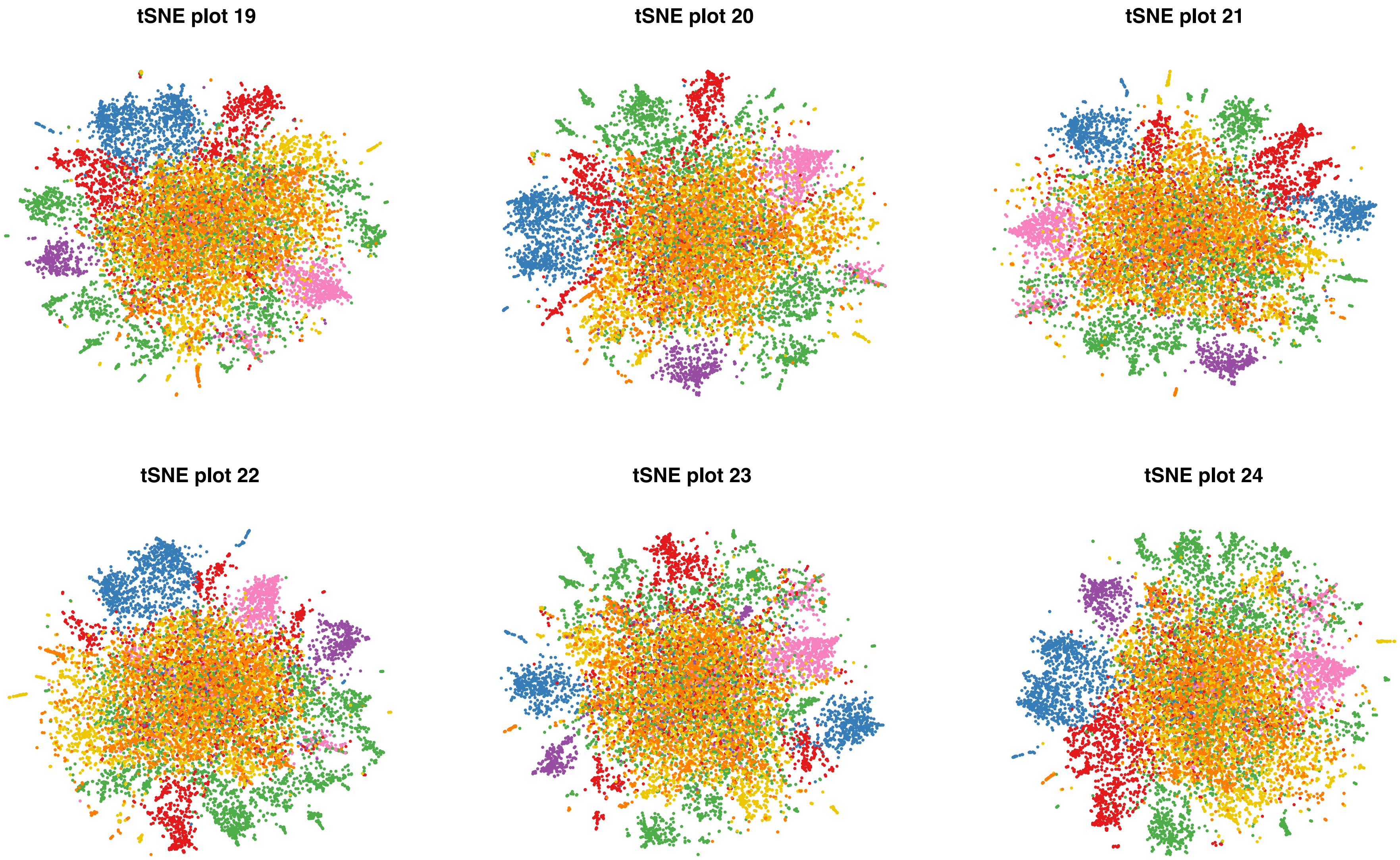
tSNE plot 19
tSNE plot 20
tSNE plot 21
tSNE plot 22
tSNE plot 23
tSNE plot 24

tSNE plot 25

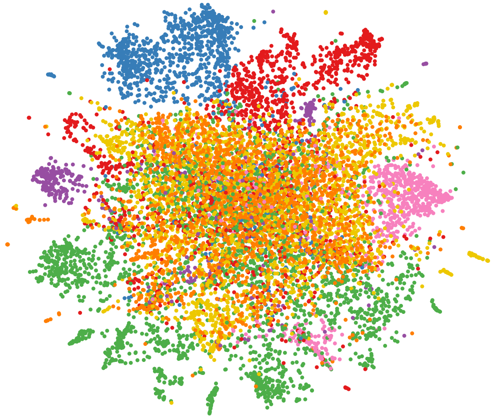

tSNE plot 26

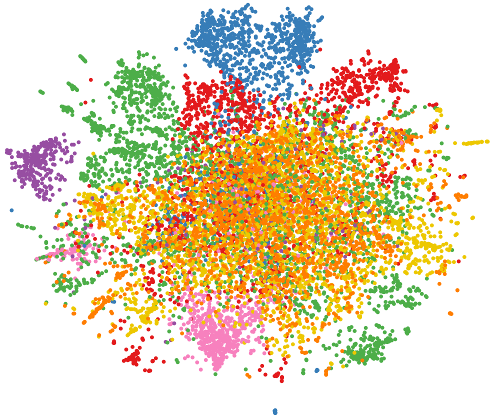

tSNE plot 27

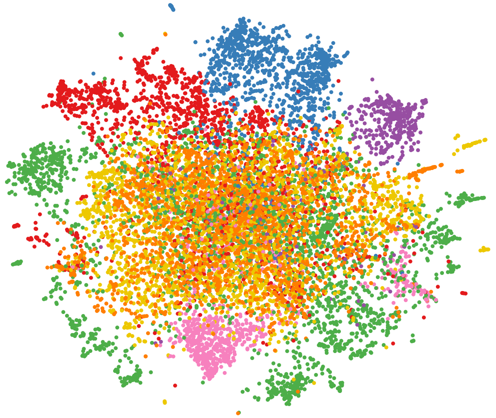

tSNE plot 28

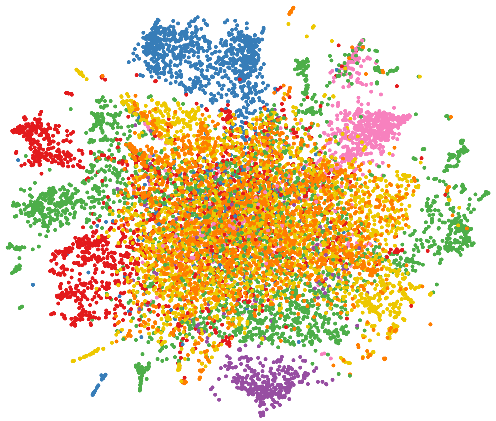

tSNE plot 29

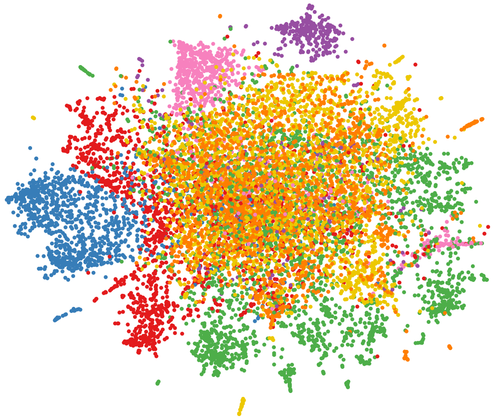

tSNE plot 30

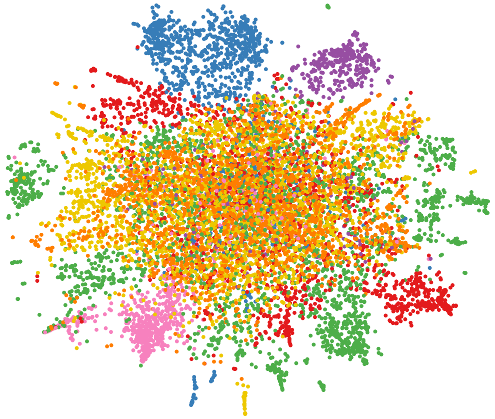

Supplement: Supplementary file 1 — Additional file 1: S1 Fig. Replicated t-SNE plots. A total of 30 replicate t-SNE plots similar to Fig. 1 were produced using different random seeds. See Fig. 1 for details. The reproducible representation of specific gene sets can be explored using the web interface at https://vigilab.shinyapps.io/anopheles/. [file 12864_2021_7722_MOESM1_ESM.pdf]
