# Supplementary material for: Unravelling population structure heterogeneity within the genome of the malaria vector Anopheles gambiae
Source: BMC Genomics. 2021 Jun 8;22:422. doi: 10.1186/s12864-021-07722-y (PMC8185951; doi:10.1186/s12864-021-07722-y)
Supplement: Supplementary file 2 — Additional file 2: S2 Fig. Local consistency measures. Ranked distribution of the number of unique nearest neighbors for each gene. Three levels were used for nearest neighbours (5, 20 and 50) in order to measure consistency in small and large clusters. Genes with low values reflect higher consistency of the local neighborhood in the t-SNE plot. S3 Fig. Global consistency measures. Ranked distribution of the mean and median values of t-SNE coordinates distances between each gene and all other genes. Genes with low values reflect higher consistency of global arrangement in the t-SNE plot. S4 Fig. Ranked distribution of individual country FST values. Population FST values calculated for each country/population vs. all other individuals. S5 Fig. Ranked distribution of broad geographic regions FST values. FST values between grouped countries in East, Central and West Africa. S6 Fig. Representation of the most significant gene function over-representation clusters. Coloured lines define the clusters within which a GO term was found enriched. The three GO categories: biological process, molecular function and cellular component, are analysed separately. S7 Fig. Additional t-SNE plots for varied perplexity values. Scatter plots of t-SNE using perplexity equals 50, 100, 250 and 1000. [file 12864_2021_7722_MOESM2_ESM.pdf]

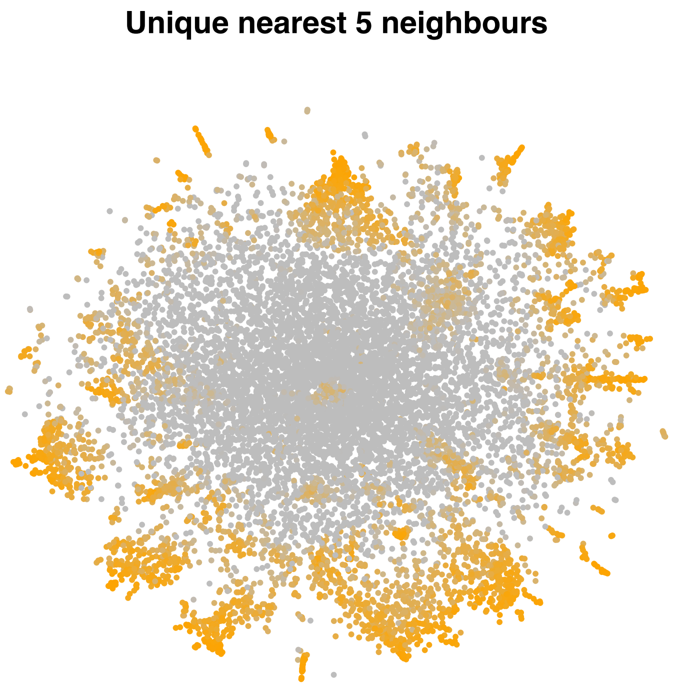
Unique nearest 5 neighbours
Unique nearest 20 neighbours
Unique nearest 50 neighbours

S2 Fig

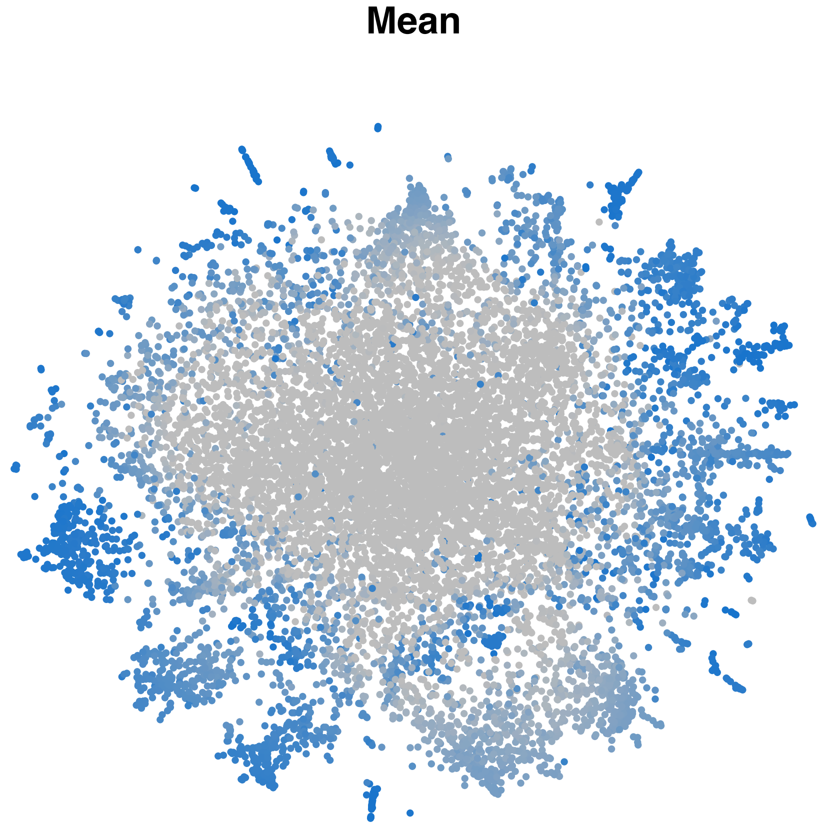

S3 Fig

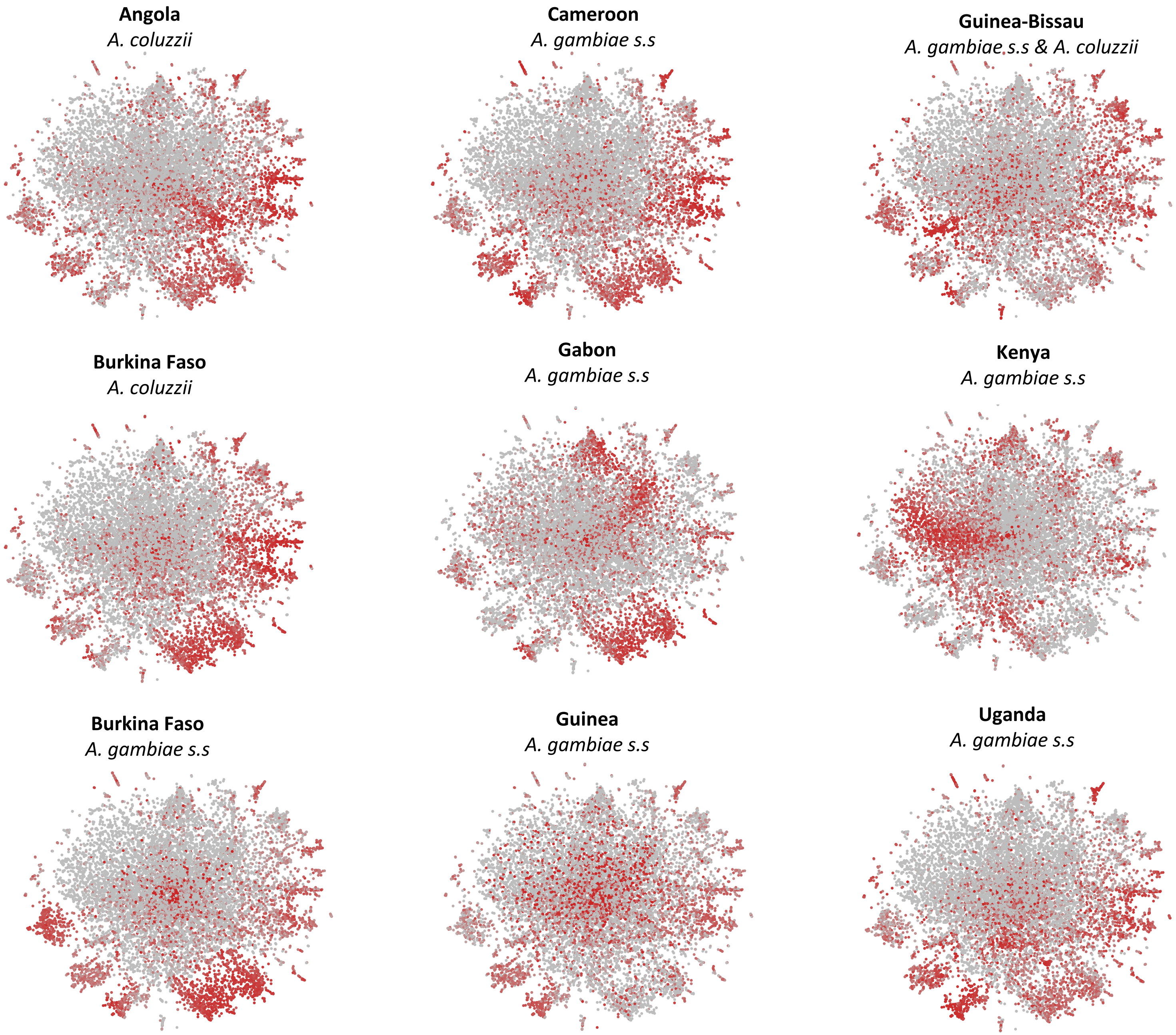
Angola
A. coluzzii
Cameroon
A. gambiae s.s
Guinea-Bissau
A. gambiae s.s & A. coluzzii
Burkina Faso
A. coluzzii
Gabon
A. gambiae s.s
Kenya
A. gambiae s.s
Burkina Faso
A. gambiae s.s
Guinea
A. gambiae s.s
Uganda
A. gambiae s.s

S4 Fig

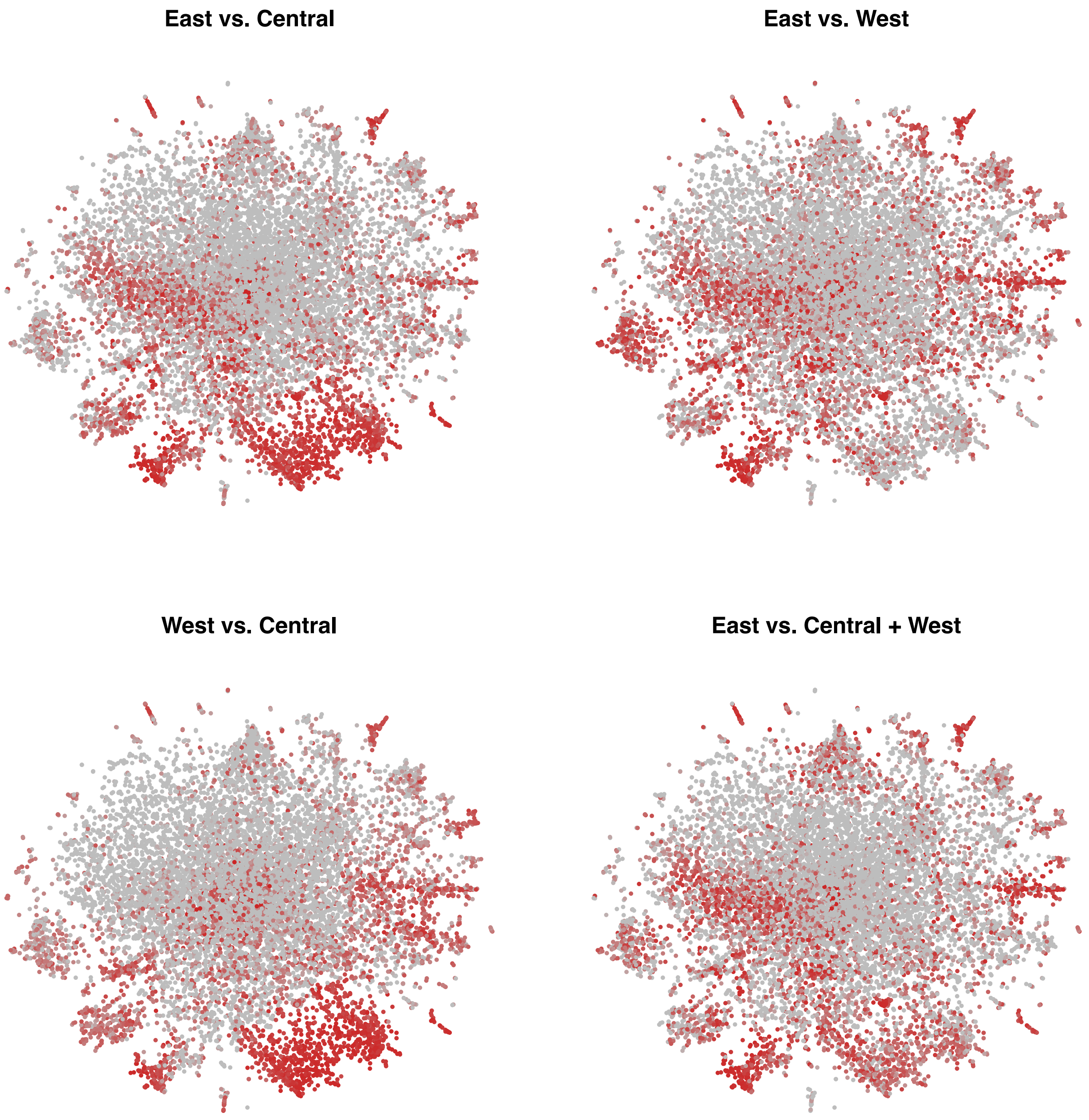
East vs. Central
East vs. West
West vs. Central
East vs. Central + West

S5 Fig

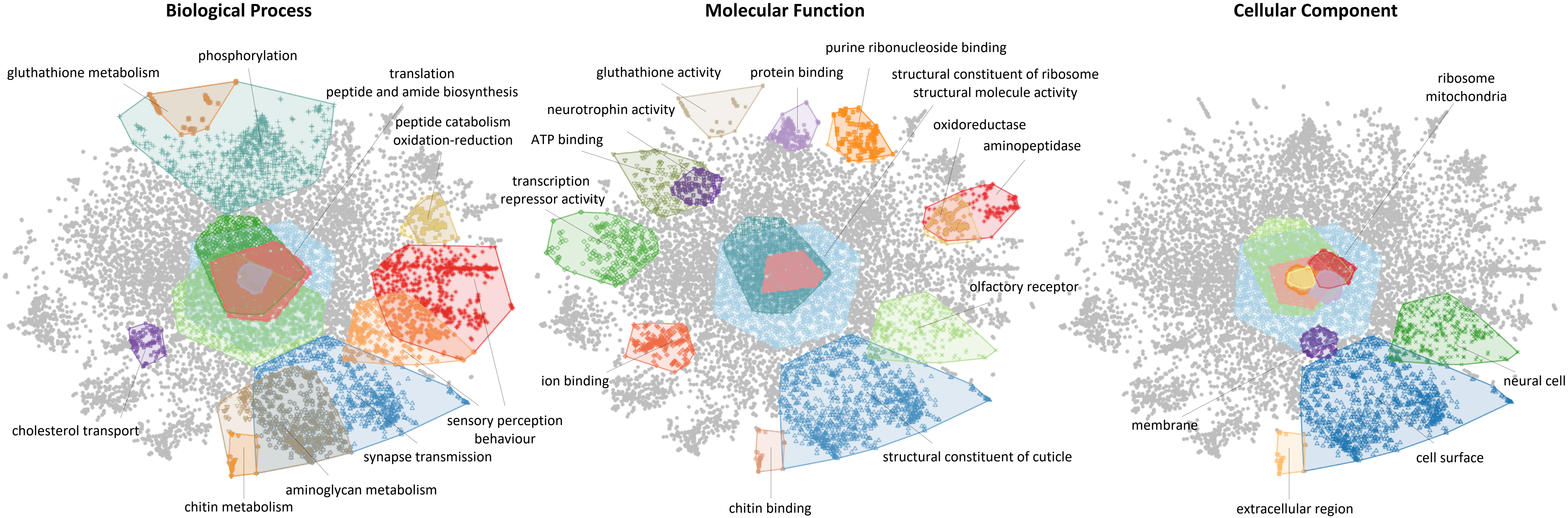
Biological Process
Molecular Function
Cellular Component
phosphorylation
gluthathione metabolism
translation
peptide and amide biosynthesis
peptide catabolism
oxidation-reduction
cholesterol transport
sensory perception
behaviour
synapse transmission
aminoglycan metabolism
chitin metabolism
purine ribonucleoside binding
gluthathione activity
protein binding
structural constituent of ribosome
structural molecule activity
neurotrophin activity
oxidoreductase
ATP binding
aminopeptidase
transcription
repressor activity
olfactory receptor
ion binding
structural constituent of cuticle
chitin binding
ribosome
mitochondria
neural cell
membrane
cell surface
extracellular region

S6 Fig

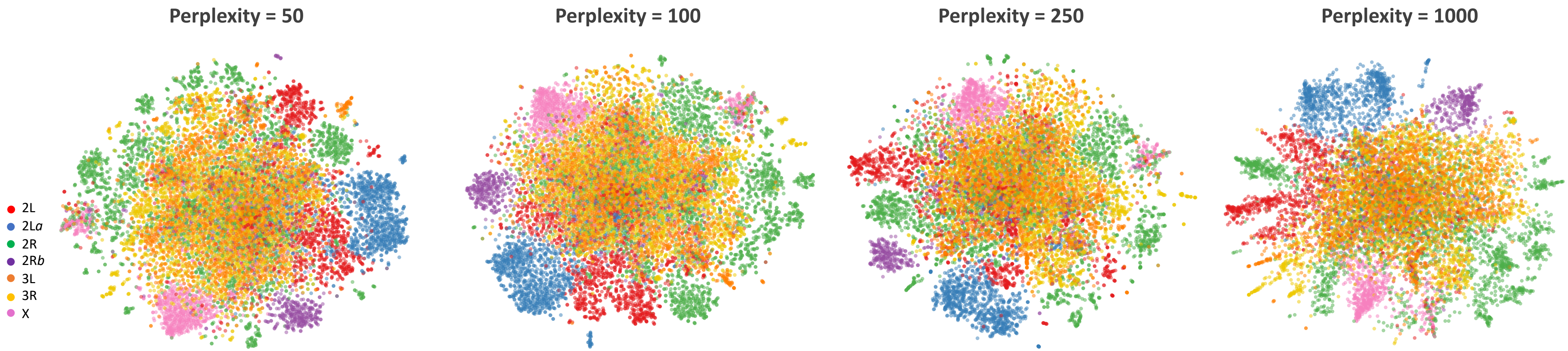
Perplexity = 50
Perplexity = 100
Perplexity = 250
Perplexity = 1000
2L
2La
2R
2Rb
3L
3R
X

S7 Fig
